# Unveiling the Potent Antiviral and Antioxidant Activities of an Aqueous Extract from *Caesalpinia mimosoides* Lamk: Cheminformatics and Molecular Docking Approaches

**DOI:** 10.3390/foods13010081

**Published:** 2023-12-25

**Authors:** Anuwatchakij Klamrak, Jaran Nabnueangsap, Jaraspim Narkpuk, Yutthakan Saengkun, Piyapon Janpan, Napapuch Nopkuesuk, Arunrat Chaveerach, Samaporn Teeravechyan, Shaikh Shahinur Rahman, Theerawat Dobutr, Poramet Sitthiwong, Pornsuda Maraming, Natsajee Nualkaew, Nisachon Jangpromma, Rina Patramanon, Sakda Daduang, Jureerut Daduang

**Affiliations:** 1 Division of Pharmacognosy and Toxicology, Faculty of Pharmaceutical Sciences, Khon Kaen University, Khon Kaen 40002, Thailand; anuwat_kla@yahoo.com (A.K.); yutthakan_s@kkumail.com (Y.S.); j.piyapon@kkumail.com (P.J.); napapuch.aom25@gmail.com (N.N.); shahinanft@gmail.com (S.S.R.); aoimacth@me.com (T.D.); nnatsa@kku.ac.th (N.N.); 2 Protein and Proteomics Research Center for Commercial and Industrial Purposes (ProCCI), Khon Kaen University, Khon Kaen 40000, Thailand; pornsma@kku.ac.th (P.M.); nisaja@kku.ac.th (N.J.); narin@kku.ac.th (R.P.); 3 Salaya Central Instrument Facility RSPG, Research Management and Development Division, Office of the President, Mahidol University, Nakhon Pathom 73170, Thailand; jaran.nab@mahidol.edu; 4 Virology and Cell Technology Research Team, National Center for Genetic Engineering and Biotechnology (BIOTEC), National Science and Technology Development Agency (NSTDA), Pathumthani 12120, Thailand; jaraspim.nar@biotec.or.th (J.N.); samaporn.tee@biotec.or.th (S.T.); 5 Department of Biology, Faculty of Science, Khon Kaen University, Khon Kaen 40002, Thailand; raccha@kku.ac.th; 6 Department of Applied Nutrition and Food Technology, Faculty of Biological Sciences, Islamic University, Kushtia 7000, Bangladesh; 7 Khaoyai Panorama Farm Co., Ltd., 297 M.6, Thanarat Rd., Nongnamdang, Pakchong, Nakhonratchasima 30130, Thailand; poramet.sitthiwong@gmail.com; 8 Centre for Research and Development of Medical Diagnostic Laboratories, Faculty of Associated Medical Sciences, Khon Kaen University, Khon Kaen 40002, Thailand; 9 Department of Biochemistry, Faculty of Science, Khon Kaen University, Khon Kaen 40000, Thailand

**Keywords:** *Caesalpinia mimosoides* Lamk, aqueous extract, gallic acid, antioxidant, antivirus, cheminformatics, molecular docking

## Abstract

Our group previously demonstrated that *Caesalpinia mimosoides* Lamk exhibits many profound biological properties, including anticancer, antibacterial, and antioxidant activities. However, its antiviral activity has not yet been investigated. Here, the aqueous extract of *C. mimosoides* was prepared from the aerial parts (leaves, stalks, and trunks) to see whether it exerts anti-influenza (H1N1) effects and to reduce the organic solvents consumed during extraction, making it a desirable approach for the large-scale production for medical uses. Our plant extract was quantified to contain 7 g of gallic acid (GA) per 100 g of a dry sample, as determined using HPLC analysis. It also exerts potent antioxidant activities comparable to those of authentic GA. According to untargeted metabolomics (UPLC-ESI(-)-QTOF-MS/MS) with the aid of cheminformatics tools (MetFrag (version 2.1), SIRIUS (version 5.8.3), CSI:FingerID (version 4.8), and CANOPUS), the major metabolite was best annotated as “gallic acid”, phenolics (e.g., quinic acid, shikimic acid, and protocatechuic acid), sugar derivatives, and dicarboxylic acids were deduced from this plant species for the first time. The aqueous plant extract efficiently inhibited an influenza A (H1N1) virus infection of MDCK cells with an IC_50_ of 5.14 µg/mL. Of equal importance, hemolytic activity was absent for this plant extract, signifying its applicability as a safe antiviral agent. Molecular docking suggested that GA interacts with conserved residues (e.g., Arg152 and Asp151) located in the catalytic inner shell of the viral neuraminidase (NA), sharing the same pocket as those of anti-neuraminidase drugs, such as laninamivir and oseltamivir. Additionally, other metabolites were also found to potentially interact with the active site and the hydrophobic 430-cavity of the viral surface protein, suggesting a possibly synergistic effect of various phytochemicals. Therefore, the *C. mimosoides* aqueous extract may be a good candidate for coping with increasing influenza virus resistance to existing antivirals.

## 1. Introduction

Medicinal plants are potential sources of bioactive ingredients used for various purposes, including the treatment of infectious and non-infectious diseases [1,2,3]. Natural plant extracts may provide healing efficacy and patient safety; however, the search for alternative phytochemical constituents with antiviral activity is still relatively limited compared to the search for antibacterial agents [1,3,4]. Thankfully, numerous studies have demonstrated the potential of aqueous and/or ethanolic extracts of plants as promising sources of antiviral agents [5,6,7,8]. Most of them are polyphenols, including hydroxybenzoic acids (e.g., gallic acid (GA) (1) and vanillic acid), hydroxycinnamic acids (e.g., caffeic acid, ferulic acid, and *p*-coumaric acid), and phenylpropanoid pathway-derived products (e.g., quercetin, kaempferol, cyanidin, resveratrol, and catechin) [9,10,11]. Antiviral activity can be detected by inhibiting viral infection during steps such as attachment, entry, replication, and particle maturation [12]. A recent study showed that the aqueous extract of *Toona sinensis*, containing catechin and GA (1) as major phenolic compounds, exerts potent antiviral activity against the influenza (H1N1) virus by blocking viral attachment to A549 cells [12,13], specifically by interacting with the viral surface proteins neuraminidase (NA) and hemagglutinin (HA). Ellagic acid (2), the dimeric structure of GA (1), present as a major compound in the aqueous extract of *Lagerstroemia speciosa*, was able to inhibit replication of human rhinovirus-4 (HRV-4) in HeLa cells [14]. Polyphenols derived from *Aronia melanocarpa*, such as isoquercetin, kaempferol, ferulic acid, caffeic acid, ellagic acid, and myricetin, have also been shown to have promising anti-influenza activities, with the latter two examples improving the survival rate of mice infected with the rPR8-GFP virus [15,16,17]. Although polyphenols are widely distributed in various plant families, the plant origin and the technological approaches restrict the large-scale production for antiviral applications, including recovery, extraction, and purification [18,19,20]. Therefore, identifying the plants with high levels and varieties of the aforementioned substances (e.g., GA and other derivatives) is of primary importance.

*Caesalpinia mimosoides* Lamk is an edible plant belonging to the *Fabaceae* family and is known by a variety of local Thai names such as cha rueat (general), thanao song, phak puya, nam puya, phakkat ya, phak khaya, and phak khaya (http://www.qsbg.org/Database/plantdb/mdp/medicinal-specimen.asp?id=352, accessed on 31 January 2023). In Asian countries, its young shoots and leaves are consumed as fresh or cooked vegetables [21,22]. Historically, this plant has been utilized as a carminative and to heal dizziness and fainting, indicating its potential safety and capacity as a medicinal agent. *C. mimosoides* Lamk exhibits promising biological activities, including anticancer, antibacterial, antioxidant, anti-inflammatory, and neuroprotective effects [23,24,25,26,27,28,29]. While the virucidal effects of this plant species have not yet been investigated, previous studies revealed that *C. minax* and *C. latisiliqua*, which belong to the same genus, were rich in flavonoids, stilbene, phenolics, and other bioactive constituents and demonstrated promising antiviral activity [30,31]. Remarkably, our group demonstrated that the *n*-hexane extract of this plant possesses anticancer properties against cervical carcinoma cell lines, but the active constituents were still unclear [27]. After partial purification using column chromatography, the most active fraction (F21) consisted of GA (1) (68.49%), ferulic acid (20.63%), vanillic acid (5.59%), caffein (3.44%), and resveratrol (1.85%). These phenolic substances, especially GA (1) as the major constituent, were speculated to play a significant role in the cytotoxic effects on cancer cells. Later, GA (1) was successfully isolated and purified; it was found capable of inhibiting the proliferation of cancer and pathogenic bacterial cells [32]. Although *C. mimosoides* contains several polyphenols corresponding to antiviral compounds reported in the literature, its antiviral activity has not yet been documented. While phenolics can be extracted from this plant species, the procedures are rather complicated and costly, as large amounts of organic solvents (e.g., *n*-hexane, MeOH, and EtOAc) are required. Hence, there is great interest in preparing *C. mimosoides* phenolic-rich extracts with antiviral activity using a green chemistry approach, enabling large-scale production to serve pharmaceutical use.

Apart from NMR, high-resolution LC-MS/MS is one of the most important tools in metabolomics analysis through its remarkable sensitivity and capacity to identify the broadest range of metabolites present in biological samples via untargeted metabolomics analysis [33,34,35,36]. Several tools are now designed for the structural annotation of natural products based on experimental mass data [37,38,39,40]. SIRIUS is a mass spectra prediction tool developed to provide the correct molecular formula and fragmentation pathway of the query subject using high-resolution isotopic pattern analysis [38]. This software also integrates with other tools supporting structure elucidation, including the CSI:FingerID web service and CANOPUS (class assignment and ontology prediction using mass spectrometry), enabling the elucidation of possible candidate structures, substructures, and classifications of the query metabolites [37,39]. For example, Wang et al. [41] recently published structural information on phytochemicals in *Salvia miltiorrhiza* Bunge, which is utilized in traditional Chinese medicine for treating neurological and cardiovascular diseases, using SIRIUS (version 4) in conjunction with mass spectrometry fragmentation rules. A total of 34 tanshinones, 31 phenolics, and 2 new salvianolic acids (salvianolic acid C and 7″,8″-Dedihydro-salvianolic acid B) were deduced from their untargeted metabolomics data for the first time [42]. Similarly, SIRIUS 4 contributed to the structural annotation of 7 unknown and 17 known bioactive constituents present in the crude extract of *Urtica dioica* leaves [43]. Thus, knowing the structural details of various compounds found in the aqueous extract of *C. mimosoides* annotated using the cheminformatics approaches would be extremely helpful for explaining mechanisms underlying parts of its radical scavenging and antiviral activities.

**Figure 1:**
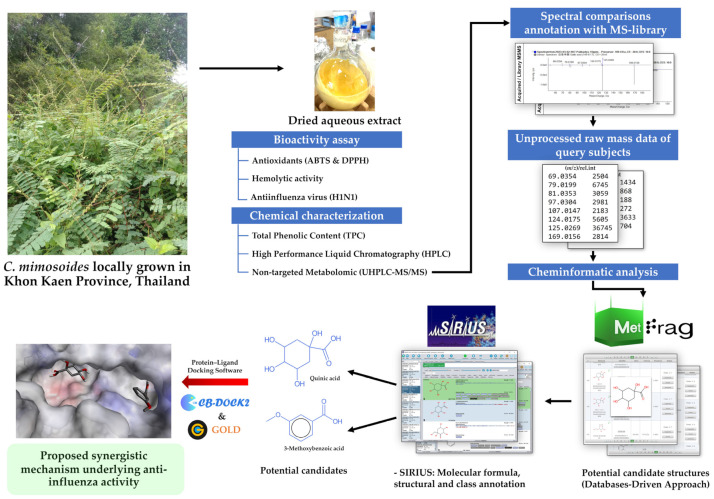
An infographic illustrating sample preparation, chemical characterization, cheminformatics-based structure annotation, antiviral assay, and molecular docking of the *C. mimosoides* aqueous extract. Structural annotation and docking of putative quinic acid (*m*/*z* 191.0607 [M-H]^−^) and 3-Methoxybenzoic acid (*m*/*z* 151.0402 [M-H]^−^) were chosen as the examples in the workflow diagram.

Here, an aqueous extract of *C. mimosoides* was established from its aerial parts, comprising leaves, stalks, and trunks, gathered from an open field in Khon Kaen province, Thailand. After an assessment of total phenolic content (TPC) and antioxidant properties, HPLC analyses were used to determine whether GA (1) was the major constituent in the extract. Untargeted metabolomics analysis (UPLC-ESI(-)-QTOF-MS/MS) was carried out to gain a better understanding of the phytochemicals in the extract, and structural information was then annotated in the coherent assessment of cheminformatics tools, including MetFrag, SIRIUS, CSI:FingerID, and CANOPUS. The anti-influenza (H1N1) activity of the *C. mimosoides* extract was evaluated, while molecular docking was employed to elucidate the potential mechanisms responsible for its antiviral activity. The infographic describing an overview of the experiments is shown in Figure 1 below.

## 2. Materials and Methods

### 2.1. Plant Collection and Preparation of C. mimosoides Aqueous Extract

Fresh aerial *C. mimosoides* parts, including leaves, stalks, and trunks, were collected from an open field at Nam Phong subdistrict, Khon Kaen province, Thailand, which is located at 16°37′56.7″ north latitude, and 102°46′14.4″ east longitude. Collected plant materials were washed in running water (30–45 min), soaked in tap water (60 min), and finally rinsed with deionized water to remove unwanted residues. Cleaned plant samples were dried at 50 °C for 2–3 days or until constant weight was reached using an oven incubator (BINDER Inc., Bohemia, NY, USA). Oven-dried plant materials were cut into smaller sizes and ground in a blender. To prepare the aqueous extract, dried finely ground plant material (20 g) was extracted with 200 mL of deionized water with shaking at 180 rpm, 25 °C for 2 days in an incubator shaker (N-BIOTEK, Gyeonggi-do, Republic of Korea). The clear supernatant was collected via centrifugation at 25 °C, 8000 rpm for 20 min, decanted into a round-bottom flask, and rotated in a cryo-bath (CoolSafe, Lynge, Denmark) at −80 °C for 1 h. Samples were then freeze-dried at −110 °C (CoolSafe, Denmark) and collected after 48 h. The freeze-dried extract was kept at −80 °C until used.

### 2.2. Total Phenolic Content (TPC)

The total phenolic content of the *C. mimosoides* aqueous extract was determined following Folin and Ciocalteu [44] with minor modifications [45]. In brief, the reaction was assessed in a 96-well plate, where an aliquot (20 µL) of extract, purified GA (1) (>95% purity Sigma-Aldrich, St. Louis, MO, USA) or blank (deionized water) was loaded in each well. Then, 100 μL of a 0.2 M Folin–Ciocalteu reagent and 80 μL of 7% (*w*/*v*) sodium carbonate were added. Mixtures were incubated at ambient temperature under constant darkness for 30 min. The absorbance value was measured at 760 nm using a microplate reader (Ensight^®^ Multimode Plate Reader, PerkinElmer, MA, USA). All detected phenolics were represented as GA equivalents (GAE)/mg dry weight.

### 2.3. Antioxidant Assays

The 2,2-diphenyl-1-picrylhydrazyl (DPPH) and 2,2′-azinobis-3-ethylbenzotiazoline-6-sulfonic acid (ABTS) assays were used to investigate the radical scavenging activity of the *C. mimosoides* water extract as recently described by Xiao et al. [46] with some modifications [46]. Purified GA was used as the reference standard, and deionized water was the blank throughout the study.

For the DPPH assay, various concentrations of the extract were mixed with 100 μL of a 0.2 mM DPPH reagent and incubated for 30 min under darkness. The reducing power of phytochemicals was evaluated by monitoring the decreased absorbance at 517 nm using a microplate reader (Ensight^®^ Multimode Plate Reader). Results were expressed as IC_50_ or the percentage of DPPH radical scavenging activity, which was calculated as follows: inhibition ratio (%) = (A_control_ − A_sample_) × 100/A_control_, where A_control_ = absorbance of the reaction with deionized water and A_sample_ = absorbance of the reaction with extract solution.

For the ABTS assay, 200 μL of an ABTS working solution was mixed with 10 μL of different concentrations of the extract, incubated at room temperature, and protected from light for 7 min. Decolorization was measured at 734 nm using a microplate reader (Ensight^®^ Multimode Plate Reader). Results were expressed as IC_50_ and/or the percentage of radical scavenging activity, which was calculated according to the formula: % free radical scavenging = (A_control_ − A_sample_) × 100/A_control_, where A_control_ = absorbance of the reaction with deionized water and A_sample_ = absorbance of the reaction with extract solution.

### 2.4. HPLC Analysis of GA (1) in the C. mimosoides Aqueous Extract

Reversed-phase high-performance liquid chromatography with a diode array detector (RP-HPLC-DAD) was used to estimate the amount of GA (1) present in the aqueous extract, as previously established by Palasap et al. [27], with slight modifications. The analysis was conducted using HPLC (1260 Infinity II LC System, Agilent, CA, USA) coupled with a PDA detector. A C-18 column (100 mm × 4.6 mm, 5 µm particle size, Phenomenex, CA, USA) was employed. The mobile phases consisted of 0.5% phosphoric acid (solvent A) and methanol (solvent B). Separation was achieved using a linear gradient of solvent B as follows: 5% for 0–2 min, 5–95% for 15 min, 95% for 3 min, and back to 5% for 10 min, through a total run time of 20 min with a flow rate at 0.8 mL/min. The sample temperature was controlled to 25 °C. Column oven temperature was controlled at 25 °C, and chromatograms were recorded at 270 nm. The sample (final concentration of 1 mg/mL) was prepared by reconstituting 10 mg of *C. mimosoides* powder extract in 10 mL deionized water, centrifuging at the maximum speed (18,894× *g*, 25 °C, 10 min), and filtering through a polyvinylidene difluoride membrane (Agela Technologies, Wilmington, DE, USA). Purified GA in deionized water (1 mg/mL) was used to generate linear 6-point calibration curves (15.625–500 μg/mL) of the standard. The experiment was run in triplicate.

### 2.5. Detection of Metabolites Using LC-MS/MS

A non-targeted metabolomic (LC-MS/MS) approach was employed to gain more details concerning the phytochemicals in the *C. mimosoides* aqueous extract. Parameters were based on Wu et al. [34], with appropriate modifications [27]. The analysis was implemented using the ultrahigh-performance liquid chromatograph (UHPLC) (UltiMate 3000 RSLCnano UHPLC System, Thermo Scientific, Waltham, MA, USA), which was equipped with an Acclaim^®^ RSLC120 C18 (100 × 2.1 mm, 2.2 µm 120 Å, Thermo Scientific, USA) column. The mobile phases consisted of 0.1% formic acid (solvent A) and 0.1% formic acid in acetonitrile (solvent B). Elution was performed at 0.3 mL/min using a linear gradient of the solvent B as follows: 0% for 0–1 min, 0–25% for 1–3 min, 95% for 3–4.5 min, and back to 0% for 2.5 min, for a total run time of 7 min. The one μL of 10 ppm *C. mimosoides* aqueous extract was passed through the column at a temperature of 30 °C. Metabolite identification was confirmed using a mass spectrometer (TripleTOF6600+, AB SCIEX™, Framingham, MA, USA). ESI source conditions were set as follows: the ion source gas 1 50, ion source gas 2 60, curtain gas 30, a temperature of 500 °C, with the ion spray voltage floating of −4500 V. A TOF MS scan range was 100–500 amu., where the product ion scan range was 40–200 Da. The scan accumulation time was 0.1 s, while the parent ion scan accumulation time was 0.25. Mass spectra were generated using a decluttering potential (DP) of −80 V via the collision energy of −30 ± 10 eV. Then, tentative identification of target metabolites was confirmed by comparing their experimental MS/MS spectra with NIST 2017 and Natural Products HR-MS/MS library containing ~13,800 and 1000 substances.

### 2.6. Structural Annotation Using MetFrag Webservice

Raw data of twenty-four metabolites detected from the extract were further annotated using the MetFrag web service. Structural annotation of the target metabolite could be accomplished via two steps of data processing, “retrieving candidates” and “fragmentation setting & processing” (accessed on 3 July 2023 at https://ipb-halle.github.io/MetFrag/projects/metfragweb/). Using the putative mass peak of GA (1) (*m*/*z* 169.0241 [M-H]^−^) as an example, by searching against the KEGG database, the neutral mass of 170.02153 (relative mass deviation 5 ppm) and its theoretical molecular ion with *m*/*z* 169.014247 were defined as parameters to receive various candidates of the query subjects. In this step, the neutral formula could also be guided. After receiving a number of candidates, other parameters, including relative mass deviation (5 ppm), absolute mass deviation (0.001), adduct type ([M-H]^−^), and MS/MS peak list (Table S1), were then provided in the fragmentation processing step to yield a ranked score of candidates along with chemical structures with linked database, exact mass, neutral formula, and matched peaks. In this case, the query subject (*m*/*z* 169.0241) was annotated as GA (score of 1.0), with 4/7 peaks matching those of in silico-generated fragments of pure GA (1) deposited in the KEGG database. Raw data of twenty-four metabolites are shown in the Supplementary Section (Table S1). Since our query subjects originated from a plant species, biological databases such as NORMAN, KEGG, and LipidMaps were chosen to evaluate the rate of correct identification (accuracy) of the natural products.

### 2.7. Structural Elucidation Using SIRIUS

Raw mass data target metabolites (18 out of 24) were further elucidated with SIRIUS (version 5.8.3). Using the putative GA (1) as an example, its raw MS^2^ data were first imported into the software. The MS^2^ level with a CID value of 35 eV was defined, the precursor ion (*m*/*z* 169.0156) and the adduct type was set, where an expected neutral formula (i.e., C_7_H_6_O_5_) could be optionally guided for a more specific formula annotation of this query subject. SIRIUS, CSI:FingerID, and CANOPUS were all selected to acquire full details, where all biological databases (e.g., KEGG, NORMAN, Plantcyc, and Natural Products) were also chosen to improve the correctness of annotation of the inquiry metabolite. By doing so, the ion *m*/*z* 169.0156 [M-H]^−^ was best annotated as “gallic acid” in multiple aspects of elemental, structural, substructural, and class annotations, consolidating those predicted by an in-house MS/HRMS library and MetFrag.

### 2.8. Anti-Influenza Virus Screening Assay

MDCK cells were seeded at 1.5 × 10^5^ cells/well in a 96-well plate in a serum-free OptiMEM (Invitrogen, Waltham, MA, USA) supplemented with 2 µg/mL TPCK-treated trypsin and incubated at 5% CO_2_ for 24 h at 37 °C. The influenza virus strain A/Puerto Rico/8/34 (H1N1) was diluted and mixed 1:1 with the extract for a final virus concentration of 100 pfu/well and a final extract concentration ranging from 1.56 to 200 μg/mL. After the cells were washed once with PBS, the virus-compound mixture was added and then incubated for an additional 24 h. Treated cells were then washed once with PBS, fixed with ice-cold acetone, and incubated at room temperature for 15 min. After washing with PBS, the cells were blocked with PBS containing 0.5% Tween 20 (PBST) and 2% BSA and incubated at room temperature for 30 min. Consequently, a 1:2000 dilution of the primary mouse anti-influenza A NP-UNLB antibody (BioLegend, San Diego, CA, USA) in PBST with 1% BSA was added to each well and incubated further for 60 min. Reactions were washed four times with PBS, followed by the addition of a 1:10,000 dilution of the secondary HRP-conjugated goat anti-mouse antibody (BioLegend) in PBST with 1% BSA and incubated for 60 min at room temperature. Signal detection was carried out by adding TMB substrate followed by incubation at room temperature for 8 min, and the signal was quantified with a microplate reader at an optical density of 450 nm. The toxicity of the extract towards MDCK cells was evaluated using the standard MTT assay as previously described [47] at concentrations ranging from 100 to 0.05 μg/mL.

### 2.9. Hemolytic Activity Assay

The hemolytic assay was established based on the hemolysis of human red blood cells (hRBCs) [48]. Extract concentrations ranged from 800 to 100 µg/mL. In brief, hRBCs were resuspended in PBS (pH 7.4) at a 4% final concentration. One hundred microliters of suspension was transferred to a new microcentrifuge tube, followed by adding 10 µL of plant extract, and the mixture was incubated at 37 °C for 60 min. After that, reactions were centrifuged at 10,000× *g* for 5 min, and the obtained supernatant was transferred into a 96-well plate and measured at 415 nm. The hemolytic activity of plant samples was calculated as follows: % hemolysis = (S/P) × 100, where S represented the absorbance of samples (e.g., extract and PBS), while P indicated the absorbance of 0.1% Triton X-100 as a positive control. Under 15% hemolytic activity signifies that the plant extract is likely safe and could possibly be applicable for human use [49].

### 2.10. Molecular Docking

The crystal structures of influenza A (H1N1) HA (ID: 1RU7; resolution 2.30 Å) and NA (ID: 6HP0; resolution: 1.88 Å) were retrieved from the protein data bank (PDB) as representative structures of these viral proteins. To find the putative binding site, cavity detection was accomplished using CB-Dock (accessed on 12 September 2023 at https://cadd.labshare.cn/cb-dock2/php/index.php), with oseltamivir acting as the query ligand. Of five candidates arranged by the binding scores, the second-rank (Vina Score of −5.4 kcal/mol) was chosen because of the specificity of the inquiry ligand towards the active site of the viral protein (Asn60, Ala62, Glu85, Ser89, Glu90, Ile93, Asp98, Phe99, Tyr102, Glu103, and Arg106) (Figure S1). Based on the basic binding of sialic acid (CID: 906), GJT (CID: 139030257), laninamivir (CID: 502272), peramivir (CID: 154234), oseltamivir (CID: 65028), zanamivir (CID: 60855), and DANA (CID: 445533) that have been found to inhibit the influenza virus via interacting with the catalytic site of NA were used as the control ligands. The GOLD 5.2.2 (Genetic Optimization of Ligand Docking) [15], incorporated installed on Intel^®^ Xeon^®^ CPU2.30 GHz, 32 CPU, Core 8 Dell server with Linux operating systems, was used for molecular docking studies following the user guide [50]. Self-docking for both HA/oseltamivir and NA/GJT complexes was conducted to ensure that their RMSD value is less than 2.0 Å; otherwise, the obtained results could be unacceptable. As predicted by the GOLD, the HA and NA received the RMSD of 1.7667 Å and 0.6478 Å, respectively, indicating both models are valid and could be openly reproduced to the inquiry metabolites from the *C. mimosoides* aqueous extract. The generic algorithm parameters of GOLD were default settings, where the target ligands were docked separately to investigate whether they could interact with the active sites of viral surface proteins as the control ligands, e.g., sialic acid and anti-neuraminidase agents. The fitness score (assigned by GOLD) defines the negative of the sum of the component energy, where the larger fitness scores are better. CB-DOCK, a web tool designed for cavity detection, protein–ligand blind docking, and homologous template fitting along with the negative Gibbs free energy assignment (kcal/mol) through the “AutoDock Vena” in the docking process (https://cadd.labshare.cn/cb-dock2/php/blinddock.php, accessed on 6 August 2023). This tool was employed to validate the acquired outcomes by uploading a part of the protein–ligand complex (in pdb format originating from GOLD docking), ensuring their complete correspondence. Hence, the receptor–ligand complexes that were subsequently generated by GOLD and CB-DOCK (version 2) were used to explain the plausible mechanisms contributing to the anti-influenza properties of the *C. mimosoides* aqueous extract throughout this study.

## 3. Results

### 3.1. Radical Scavenging Activities of the C. mimosoides Aqueous Extract

**Figure 2:**
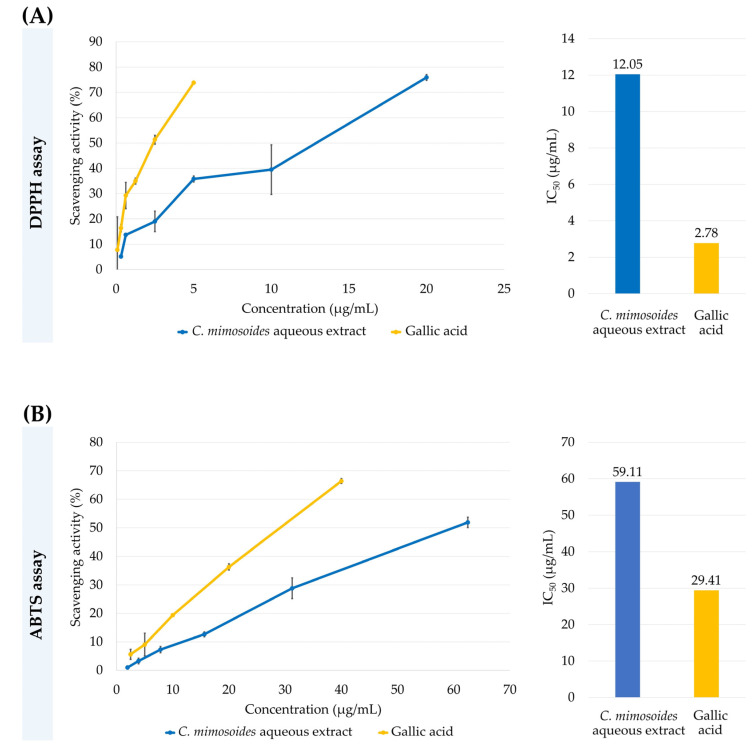
Total phenolic content and antioxidant activity of *C. mimosoides* aqueous extract. (**A**) DPPH assay; (**B**) ABTS assay. The authentic gallic acid (Sigma) was included as a positive control in all assays.

As mentioned, the ability to inhibit viral infection, especially in the case of water and ethanol extracts, is relatively straightforward for phenolic substances present in those extracts, where hydroxy groups belonging to aromatic ring systems interact with the active regions of viral surface proteins, including NA and HA [12,13]. Likewise, radical scavenging is involved in preventing cellular damage (e.g., oxidative stress) that occurs during viral entry or subsequent infection [51,52]. Thus, measuring antioxidant properties in parallel with defining the phenolic contents of extracts is a prerequisite. With the DPPH assay, the *C. mimosoides* aqueous extract exhibited potent radical scavenging activity in a concentration-dependent manner with a minimum IC_50_ value of 12.05 µg/mL, approximately 4.34-fold higher than that seen for GA (1) (2.78 µg/mL) (Figure 2A). It also scavenged the ABTS radical with IC_50_ values of 59.11 µg/mL while the GA (1) IC_50_ was 29.41 µg/mL, a 2.01-fold difference in a dose-dependent manner (Figure 2B). Notably, the obtained results provide a significant link between phenolic content (TPC) (551 mg (GAE)/g DE) (Table 1) and the plant extract’s ability to scavenge free radicals (Figure 2). This confirms our initial hypothesis that the aqueous extract of *C. mimosoides* is a viable source of phenolics with strong antioxidant properties.

### 3.2. Determination of GA (1) Using High-Performance Liquid Chromatography

**Figure 3:**
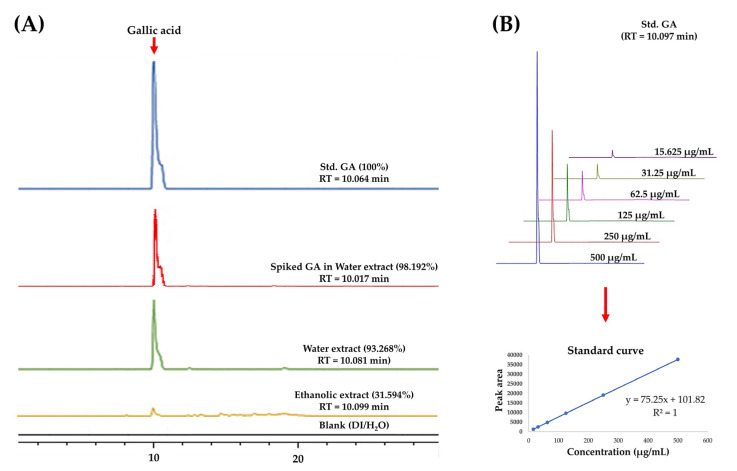
Qualitative and quantitative analysis of GA using HPLC. (**A**) Comparison of GA from different plant extracts. (**B**) Multi-point calibration curve of authentic gallic acid. The different colors represented the different samples analysed by HPLC analysis; meanwhlie, the color arrow to allocate the peaks that correponded to gallic acid, and to provide a linear correlation of the standard gallic acid.

Previous studies conducted by our group showed that GA (1) was the major constituent presented in various extracts (e.g., *n*-hexane, MeOH, and EtOAc) of *C. mimosoides* [27]. Thus, this compound was preliminarily chosen as the chemical marker for quality and quantity assessments of the *C. mimosoides* water extract using HPLC analysis. As shown in Figure 3, RP-HPLC analysis detected a significant peak (93.268% relative peak area) in the *C. mimosoides* aqueous extract, whose retention time corresponded to authentic GA (1). Also, there was a smaller peak (31.594% relative peak area) eluted at the retention time of GA (1) found in the ethanolic extract of this plant species. No metabolites corresponding with GA (1) were detected in the blank samples (H_2_O and EtOH), indicating that contamination did not occur during the sample preparation phase. To verify these results, we spiked the GA (1) standard into the *C. mimosoides* aqueous extract, which yielded only a single peak with a retention time of 10.081 min, suggesting that the major metabolite is likely to be GA (1) rather than any other phenolic substance. Quantitative analysis relying on the six-point calibration curve of GA (1) (15.625–500 μg/mL) with y = 75.25X + 101.82 (R^2^ = 1) indicated the presence of 388.05 μg/mL of GA (1) in the aqueous extract, which is equal to 3.38 mg/g DW ground plant material. The yield of GA (1) in the ethanolic extract was 0.42 mg/g DW, approximately 8.03-fold lower than that detected in the aqueous extract. Therefore, the *C. mimosoides* aqueous extract was chosen for untargeted metabolomics analysis to annotate the putative GA (1) peak and to characterize other phenolic substances that presumably govern its antioxidant and antiviral properties.

### 3.3. Metabolic Profiling of the Aqueous Extract of the C. mimosoides Using UPLC-ESI(-)-QTOF-MS/MS

**Figure 4:**
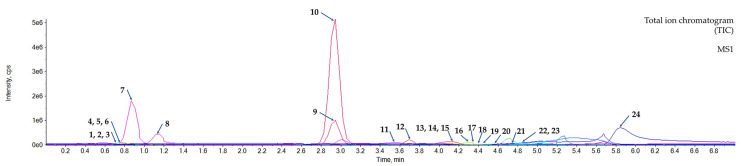
Untargeted metabolomics of *C. mimosoides* aqueous extract using negative ion mode LC-MS/MS analysis. (1) DL-Malic acid; (2) Salicylic acid; (3) Digalactouronic acid; (4) D-arabinonic acid; (5) D-sorbitol; (6) 5-keto-D-gluconic acid; (7) (-)-Quinic acid; (8) Shikimic acid; (9) Pyrogallol; (10) Gallic acid; (11) Protocatechuic acid; (12) Bergenin; (13) 4′-hydroxyacetophenone; (14) 1,2-Benzenedicarboxylic acid; (15) Benzoic acid; (16) Azelaic acid; (17) Betulinuc acid; (18) 3-Methoxybenzoic acid; (19) *trans*-Traumatic acid; (20) 3-Hydroxy-4-methoxycinnamic acid; (21) Hexadecanedioic acid; (22) Isorhamatin; (23) Tetradecanedioic acid; and (24) 3-hydroxy-4-methoxybenzoic acid.

Numerous studies have indicated that the LC-MS/MS in negative ion mode analysis typically provides much more sensitivity in creating the negative-ion species of phenolic substances present in various plant extracts rather than in positive ion mode [53,54,55]. Thus, high-resolution UPLC-ESI(-)-QTOF-MS/MS was performed, and detecting twenty-four hydroxy-containing metabolites in the *C. mimosoides* extract tentatively classified as phenolics, dicarboxylic acids, and sugar derivatives (Figure 4). As summarized in Table 2, their matching scores with those authentic spectra deposited with the in-house MS library ranged from 62.4% to 100%, with a mass error lower than 20 ppm, indicating that the obtained results were quite reliable. Remarkably, the putative GA (1) (*m*/*z* 169.0241 [M-H]^−^, Rt = 2.92 min) was detected as the major product among other substances detected (% peak area of 54.06), and its experimental fragmentation pattern was in line with the authentic GA (1) deposited in the PubChem database (accessed on 1 July 2023 at https://pubchem.ncbi.nlm.nih.gov/compound/370). This demonstrated that *C. mimosoides* could be prepared as a “GA-rich extract”, significantly reducing the number of organic solvents consumed.

### 3.4. Structural Annotation Using MetFrag

Twenty-four substances were further characterized using MetFrag. As shown in Table 3, 18 out of 24 query subjects could be structurally annotated as found in the MS library but ranked differently depending on the chosen databases. Using the ion *m*/*z* 191.0607 as an example, among 702 candidates retrieved from the PubChem database, this query subject was annotated as quinic acid (126th) with a score of 1.0 and 4/7 peaks explained, implying additional annotation using distinguished databases was required. This tool also provides various biological databases to improve structural annotation of natural products. After comparison against KEGG and NORMAN, quinic acid rose to become one of the top-ranked candidates (1st and 2nd). This inquiry subject was thus presumably quinic acid rather than its related analogs possessing an identical molecular formula (C_7_H_12_O_6_). A similar trend was found in the case of putative azelaic acid (*m*/*z* 187.0982). Its rank increased from 531st (PubChem) to first after analyzing against a lipid maps database. The six parent ions (369.0684, 193.0371, 165.0926, 455.2502, 315.2536, and 166.8340) that could not be annotated were excluded here. The remaining 18 metabolites were thus annotated using SIRIUS to their vital information regarding correct formula, structure, fingerprint, and class to fully support structural elucidation based on distinct cheminformatics approaches.

### 3.5. Structural Annotation Using SIRIUS

**Figure 5:**
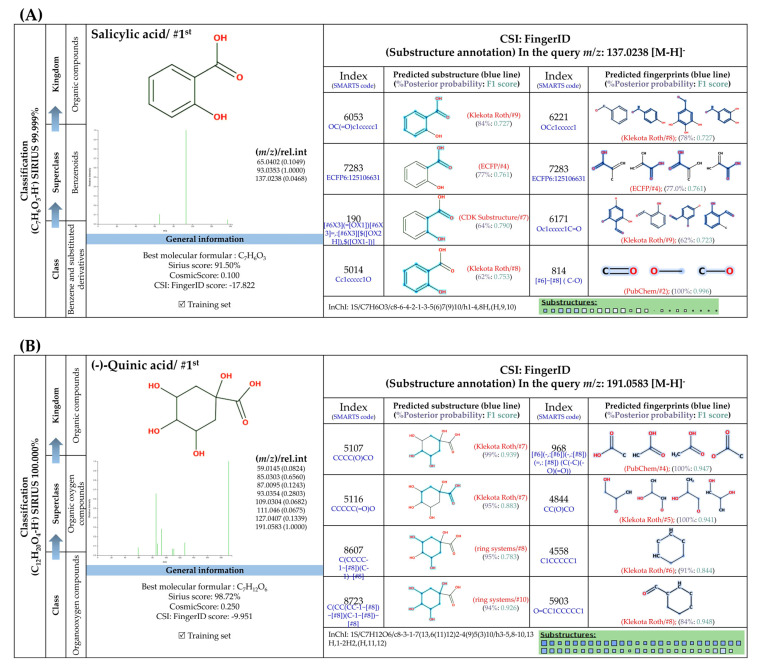

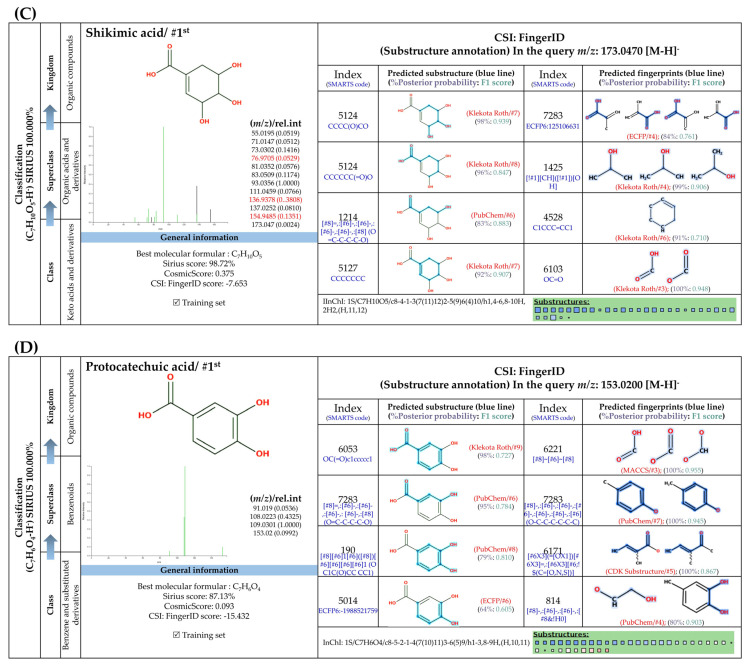

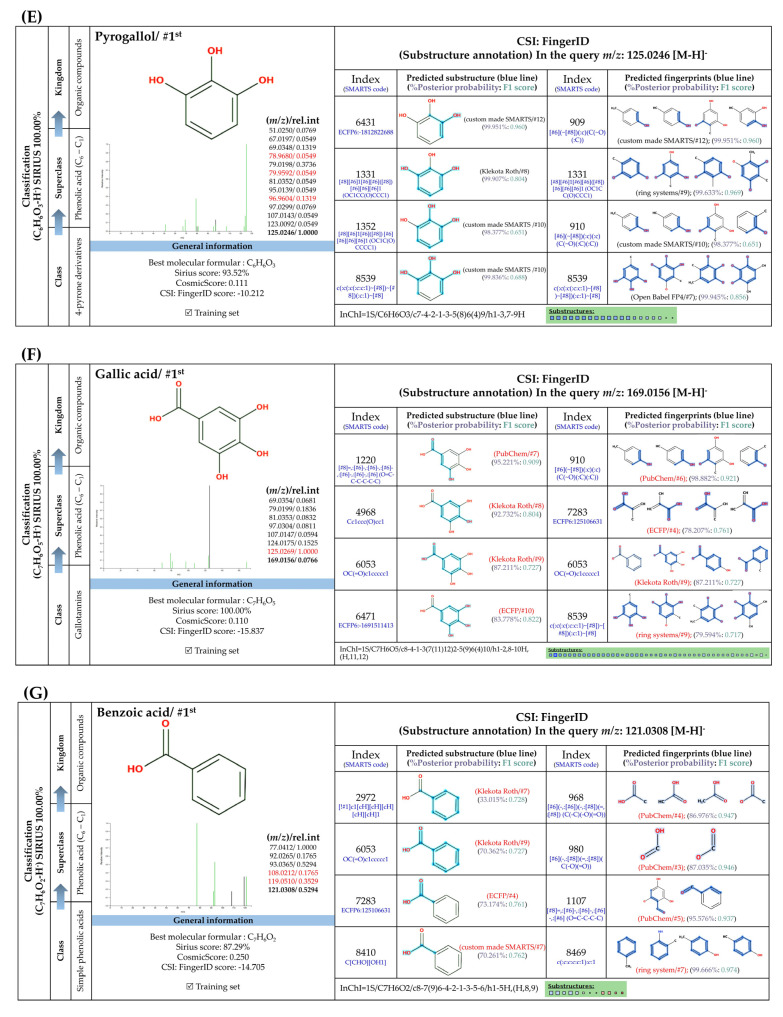
Tentative structural identification of simple phenolic acids found in the *C. mimosoides* aqueous extract using SIRIUS, CSI: FingerID, and CANOPUS. (**A**) Salicylic acid; (**B**) (-)-Quinic acid; (**C**) Shikimic acid; (**D**) Protocatechuic acid; (**E**) Pyrogallol; (**F**) Gallic acid; (**G**) Benzoic acid. All of the query subjects (**A**–**G**) were predicted to receive the correct molecular formula, based on the matched mass spectra (indicated in “green”), as the first-rank candidate with the highest SIRIUS score (87.13% to 100%). The matched tandem mass spectra (green color) were then translated into potential structures using CSI:FingerID web service, based on the pairwise comparison with those present in the biological databases (e.g., KEGG, NORMAN, and Natural Products). By taking molecular fingerprints defined by CSI:FingerID web service, CANOPUS then predicted they all share the same shikimic route and phenolic acid (C_6_-C_1_) is the superclass of the natural products.

**Figure 6:**
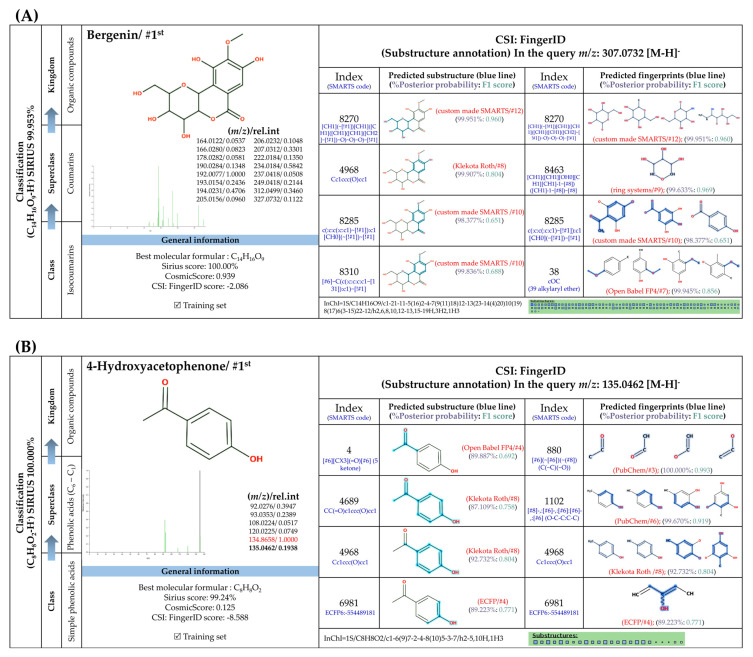

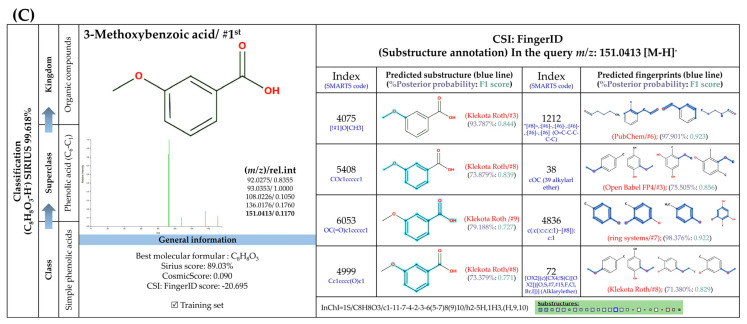
Structural annotation of putative methylated analogs synthesized via shikimate and phenylpropanoid pathway. (**A**) Bergenin; (**B**) 4-Hydroxyacetophenone; (**C**) 3-Methoxybenzoic acid. The high-resolution isotope pattern analysis illustrates the correct elemental formula of the three methylated derivatives (**A**–**C**) detected in the *C. mimosoides* aqueous extract (**A**–**C**). The matched tandem mass spectra (green color) of each metabolite were then subjected to structural annotation using CSI:FingerID web service. Numerous molecular fingerprints showing the presence of specific *O*-methylation aromatic core structures were also assigned by the CSI:FingerID web service with the highest posterior probability score (F1). CANOPUS clearly revealed that they all originated from the shikimate pathway, where phenolic acid (C_6_-C_1_) serves as the “Superclass” of these methylated derivatives.

**Figure 7:**
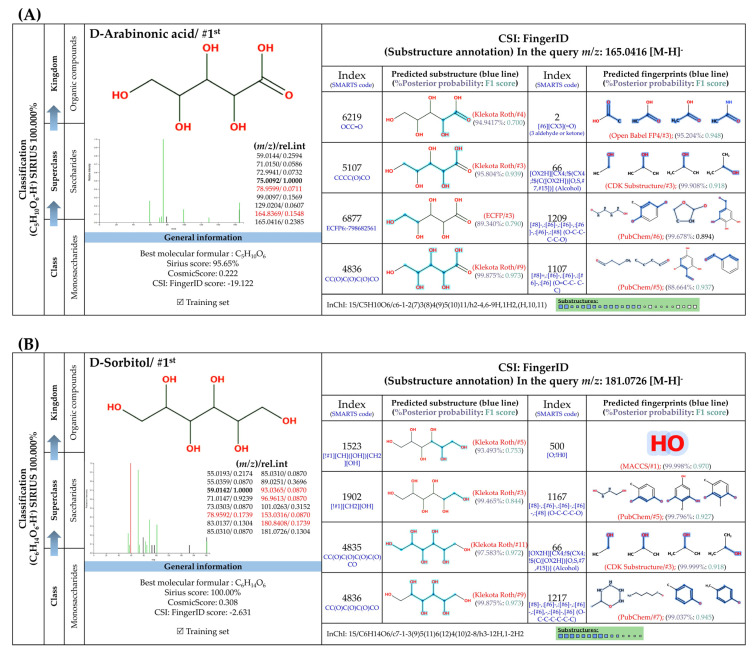
Formula, structure, and substructure annotation of sugar derivatives detected from the *C. mimosoides* aqueous extract. (**A**) D-Arabinonic acid; (**B**) D-Sorbitol. Isotope pattern analysis decodes the correct molecular formula of putative sugar derivatives (**A**,**B**) from the matched MS^2^ spectrum (showing in green color) with a SIRIUS score of 100%. The matched tandem mass spectra of each metabolite were then subjects for structural annotation using CSI:FingerID web service, where the negative ions with *m*/*z* 165.0414 and *m*/*z* 181.073 were best annotated as D-Arabinonic acid and D-Sorbitol, respectively. By taking the relevant substructures (molecular fingerprints) predicted by CSI:FingrerID web service, CANOPUS reveals that both sugars are categorized in class and subclass of saccharides, wherein “Organic compound” is a kingdom of these two natural products.

**Figure 8:**
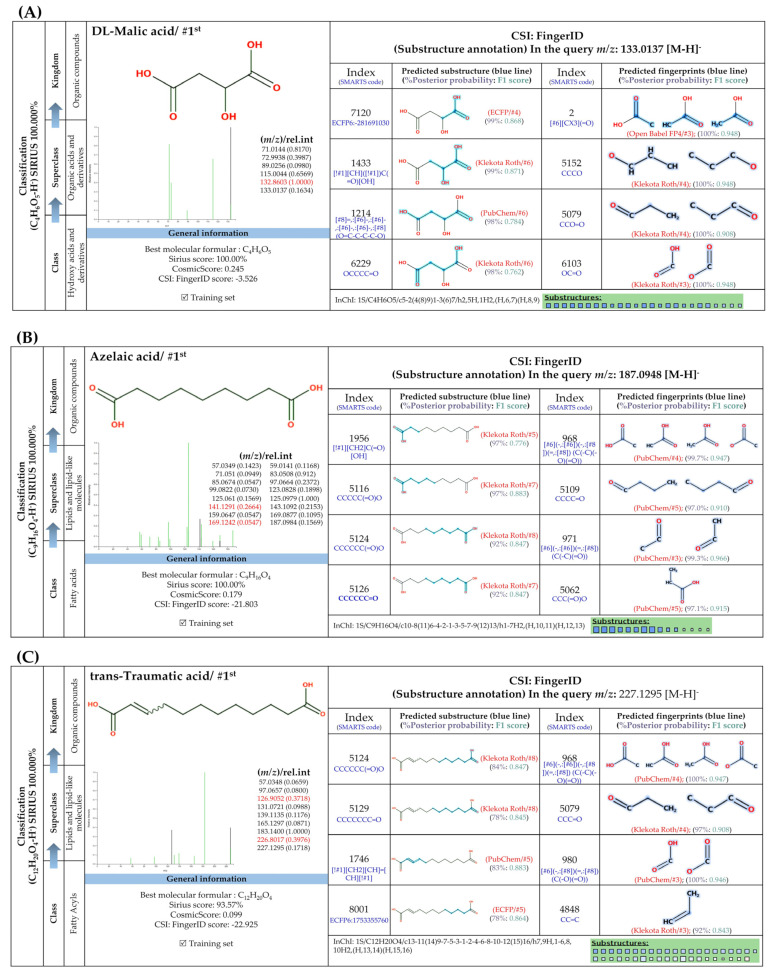

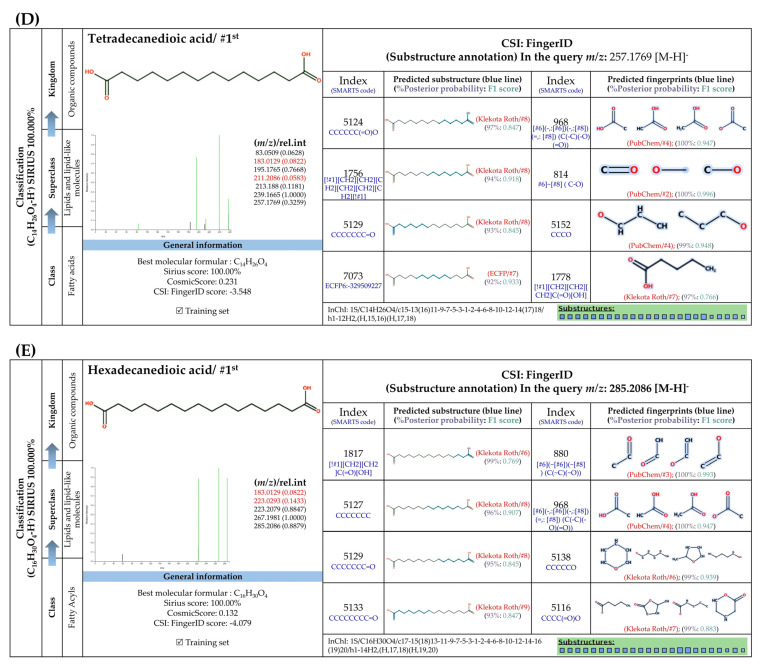
Molecular formula, structure, and substructure annotation of dicarboxylic acids detected from *C. mimosoides* aqueous extract. (**A**) DL-malic acid; (**B**) Azelaic acid; (**C**) *trans*-Traumatic acid; (**D**) Hexadecanedioic acid; (**E**) Tetradecanedioic acid. The high-resolution isotope pattern analysis unveils the correct molecular formula of putative dicarboxylic acids (**A**–**E**) that are hidden in the matched MS2 spectrum (as indicated in green) with a SIRIUS score of 100%. The green spectra of each metabolite were then translated into their highly corresponded chemical structure using CSI:FingerID web service, where numerous substructures (so-called fingerprints) that represent being of linear chain hydrocarbons and dicarboxylic acids are also detected in the query metabolites. CANOPUS, a compound classification tool, verifies that fatty acids, fatty acyls, and organic compounds are class, superclass, and kingdom of all-natural products, respectively.

SIRIUS is an in silico tool designed to annotate a correct molecular formula of inquiry subjects based on high-resolution isotope pattern analysis [38]. This software is also integrated with CSI:FingerID and CANOPUS in order to provide structure and class, fingerprints (substructures), and classes of inquiry subjects from unknown mass spectra [37,38,39]. After analysis using these tools, 17 out of 18 metabolites could be annotated to corresponding structures, as explained in Figure 5, Figure 6, Figure 7 and Figure 8. Their ranks as top candidates in terms of formula and structural annotation were remarkably high, indicating that reliable results were obtained. According to CANOPUS, they could be classified into four groups, the details of which are as follows.

#### 3.5.1. Simple Phenolic Substances

Systematic compound classification revealed that seven metabolites in this group were solely synthesized via shikimate and phenylpropanoid pathways (Figure 5). The use of high-resolution isotope pattern analysis led to unraveled-correct formulas of these phenolics, as observed from the highest SIRIUS scores (range 99.999–100%) among various formulas postulated using this tool. After searching against biological databases, CSI:FingerID confirmed that their structural information corresponded with those previously predicted by MetFrag and newly arranged as the first-ranked candidate (1st), indicating a higher reliability of the structural annotation. Several substructures (so-called “fingerprints”) that represent aromatic core structures (C_6_-C_1_), hydroxy groups, and carboxylic groups were also predicted to be present in these compounds, further supporting the precision of the predictions. The query subject at *m*/*z* 169.0156 [M-H]^−^, for example, out of the ten formulas retrieved, was best annotated as C_7_H_6_O_5_ (SIRIUS score: 100.000%), with six from seven peaks explained, which is higher than that of the MetFrag web service (4/7) (Table 3). When an expected molecular formula (C_7_H_6_O_5_) was guided during the data-filling process, the SIRIUS score reached 100%. CSI:FingerID also showed that this query metabolite (*m*/*z* 169.0156; C_7_H_6_O_5_) was annotated as GA (100% similarity; CSI score: −15.837) from among the one hundred candidates retrieved from numerous databases (Figure 5F). Several substructures of GA were detected in this query subject. For instance, fingerprint encoded by “[#8]=,:[#6]-,:[#6]-,:[#6]- ,:[#6]-, :[#6]-, :[#6]- ,:[#8] (O=C-C-C-C-C-O)” indicated intercorrelation between carbonyl group (C=O) and the hydroxy group (-OH) located on the ring system. Two substructures, encoded by “[#8][#6]1[#6][#6]([#8][#6]1 [#6][#6]1(OC1CC(O)CCC1)” and “[#8][#6]1[#6][#8][#6][#6][#6][#6]1(OC1C(O)CC CC1)”, represented the covalent linkage between the benzene ring and two oxygen atoms, while a hydroxy group (O;H0) was also deduced in the same query subject. Since pyrogallols and derivatives are alternative classes of GA, successive annotation of the six remaining metabolites was also a consequence since they share identical synthetic routes in many plant species [56,57]. This reliability of annotation might be supported by the presence of authentic mass spectra of these phenolics in CSI: FingerID training data (accessed on 20 July 2023 at https://www.csi-fingerid.uni-jena.de/v2.6/api/fingerid/trainingstructures?predictor=2). Benzoic acid (*m*/*z* 121.0663) is a notable example, where it entirely shifted to first since its data were deposited in the training database (InChI=1S/C_7_H_6_O_2_/c8-7(9)6-4-2-1-3-5-6/h1-5H,(H,8,9)).

#### 3.5.2. Methylated Analogs

O-Methylation is crucial for an increasing number of biological activities of natural products, where transferring a methyl moiety onto acceptor molecules (e.g., simple phenolic) yields methylated analogs possessing great structural diversity with improved lipophilic properties [58]. Four putative methylated phenolic derivatives, including ions at *m*/*z* 135.0822, 327.0743, 151.0402, and 193.0881, were found in the aqueous plant extract (Figure 6). With the use of high-resolution isotope pattern analysis, the expected elemental formula of these methylated metabolites was postulated according to the highest scores (ranging from 99.618 to 100%). All metabolites except an ion with *m*/*z* 193.0881 were structurally annotated as the top-ranked candidates consistent with MetFrag web service, signifying much more reliable results. Substructural annotation also revealed that the putative 3-methoxybenzoic acid (*m*/*z* 151.0402; Rt = 4.13 min) shares numerous features with its parent benzoic acid (*m*/*z* 121.0663; Rt = 4.40 min). For example, the substructure encoded as “[#8]=,:[#6]-,:[#6]-,:[#6]-,:[#6]-,:[#6](O=C-C-C-C-C)”, whose SIRIUS score of 97.901% (F1 = 0.923), illustrating positive correlation between the four carbon atoms and the carbonyl group (C=O) located in the benzene ring. They differed from each other, however, due to the presence or absence of a methyl group (-CH_3_) ether bonded (R-O-R’) with a hydroxy group on the basic ring system (C6-C1). These included C;H3,H4, cOC(39alkylaryl ether), c(:c(:c:c:c:1)~[#8]):c:1, [!#1]O[CH3], COc1ccccc1, Cc1cccc(O)c1, and [OX2](c) [CX4;!$(C([OX2])[O,S,#7,#15,F,Cl,Br,I])] (Alkylarylether). These results became more rigid by the detection of a product ion at *m*/*z* 136.0176, which illustrates an entire loss of a methyl group (~15.0238 Da) from its core structure (*m*/*z* = 151.0402). The resulting product is subject to consecutive loss of the carboxy group (CO_2_: 43.9886 Da), yielding the second-most abundant peak at *m*/*z* 92.0275 (Figure 6C). Putative bergenin *(m*/*z* 327.0743; Rt = 3.69 min) also consisted of fingerprints that illustrate successive C-glycosylation and O-methylation of GA, which could be characterized by cOC (39 al-kyl aryl ether),“[CH1]([CH1] [OH0][CH1][CH1]-1~[#8])([CH1]-1~[#8])~[#8], and [CH1](~[!#1])[CH1]([CH1]([CH1]( [CH1]( CH_2_]~[!#1])~O)~O)~O)~[!#1]. Also, substructures showing a successive pyrone ring formation between GA and a sugar moiety were detected in the same structural candidate, which could be described by [#6]~C(c(:c:c:c:c1~[131]):c1)~[!#1],Cc1ccc(O)cc1)~[!#1]):c1 [CH0](~[#1])~[!#1] and Cc1ccc(O)cc1. Compound class annotation also supported 2-benzopyrans, isocoumarins, and coumarins as alternative classes of this inquiry subject. Unfortunately, the negative ion with *m*/*z* 193.0881, formerly annotated as 3-hydroxy-4-methoxy cinnamic acid (Table 3), could not be unraveled by CSI:FingerID due to its limited fragmentation spectra. Although it contains the product ion at *m*/*z* 178.064, suggesting a methyl loss (CH_3_: 15.0238) from the cinnamic core structure, optimizing collision-induced energy is still needed to fully support the tentative identification of this methylated analog, and it was hence ruled out from this study.

#### 3.5.3. Sugar Derivatives

Two putative peaks that corresponded with sorbitol (*m*/*z* 181.073; C_6_H_14_O_6_) and D-arabinonic acid (*m*/*z* 165.0414; C_5_H_10_O_6_) were best explained by SIRIUS and the CSI:FingerID web service. Sorbitol is a reduced product of glucose that is catalyzed by aldose reductases. Substructural elucidation revealed substructures that indicate a successive reduction of glucose to the sugar alcohol detected in the first query subject. For example, fingerprints encoded by “[#8]-,:[#6]-,:[#6]-,:[#6]-,:[#8] (O-C-C-C-O)”, “[#8]-,:[#6]-,:[#6]-,:[#6]-,:[#8] (O-C-C-C-C-O)”, and “[#8]-,:[#6]-,:[#6]-,:[#6]-,:[#6],-,:[#6]-,[#6] (O-C-C-C-C-C-C)” clearly illustrate a correlation between various carbon chain lengths (C_3_-C_5_) and oxygen atoms at the termini. This is consolidated by the presence of hydroxyl group, denoted by “[OX2H][CX4;!$(C([OX2H])[O, S,#7,#15])](Alcohol)” and “[O; H0]” in this structural potential. Although polyols and primary alcohols are alternative classes of this sugar derivative, D-mannitol might also be a potential candidate due to a highly identical fragmentation profiling deposited in the PubChem database (accessed on 20 July 2023 at https://puchem.ncbi.nlm.nih.gov/#query=D-mannitol). Since both sugar alcohols are isomeric structures sharing polyol biosynthetic routes in numerous plant species [59], further elucidation is needed to support the computational-based annotation. Putative D-arabinonic acid also shares identical features to that of sorbitol, such as “[#8] -,:[#6]-,:[#6]-,:[#6]-,:[#8] (O-C-C-C-C-O)” and “[O; H0]”. Even so, they could be distinguished from one another based on particular substructures encoded by “[#8]=,:[#6]-,:[#6]-,:[#6]-,:[#6] (O=C-C-C-C)” and “[#8]=,:[#6]-,:[#6]-,:[#6] (O=C-C-C-)”, which show the presence of carboxylic group, with hydroxy acids and derivatives as ancestors of this sugar derivative.

#### 3.5.4. Dicarboxylic Acids

Short and medium-chain fatty acids (C_4_, C_9_-C_16_) were also detected and predicted to originate from fatty acid biosynthetic pathways. These included negative precursor ions with *m*/*z* 133.0149, 187.0982, 227.2021, 257.1776, and 285.2081, respectively (Figure 8). CSI: FingerID provides substructural details hidden in the query subjects. As an example, the ion at *m*/*z* 187.0892 [M-H]^−^ was annotated as “azelaic acid” as the first example. As depicted by “CCCC=O”,“CCC(=O)C”, “[#6][CX3](C=O)”, and ‘’[#8]-,:[#6]-,:[#6]-,:[#6]-,[#6]-,:[#6] (O-C-C-C-C-C), these fingerprints imply the connection between aliphatic hydrocarbons (C_3_-C_5_) and a carboxyl group. A positive correlation between hydrocarbons and carboxylic group, encoded by “[!#1][CH_2_]C(=O)[OH]” and [#6](-,:[#6])(-,:[#8])(=,:[#8] (C(-C)(-O)(=O)), was also present in the same query subject. The putative *trans*-Traumatic acid (*m*/*z* 227.2021) is distinguished from other class members via exclusive detection of the molecular fingerprint “[#6]=,:[#6] (C=C)” signifying a double bond (C=C) formation in this inquiry subject. This substructure was clearly absent in other dicarboxylic acids considering the lowest posterior probability scores (0.351–3.544%), suggesting structural annotation of the product ion at *m*/*z* 227.2021 could possibly be reliable.

### 3.6. Antiviral and Hemolytic Activity of the C. mimosoides Aqueous Extract

**Figure 9:**
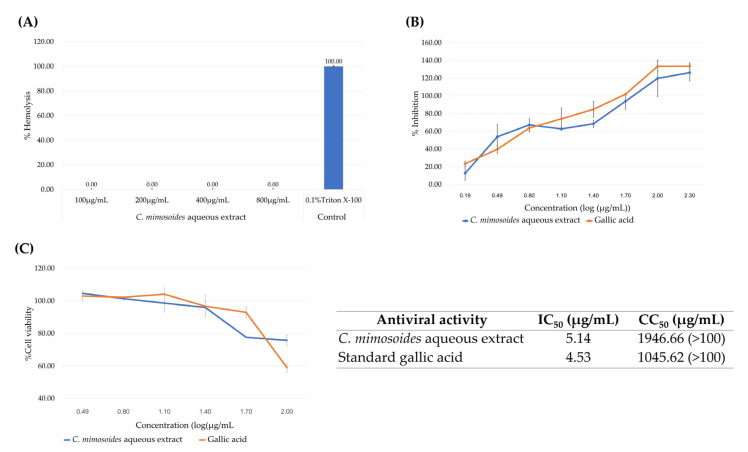
Hemolytic and antiviral activities of *C. mimosoides* aqueous extract and gallic acid. (**A**) Hemolytic activity assay; (**B**) IC_50_ defines the concentration of a substance or an extract that is required to inhibit viral replication by 50%, where the lower IC_50_ values signify a greater antiviral potency; (**C**) CC_50_ is used in the framework of cytotoxicity assays, meaning the concentration of a plant extract and/or the authentic GA (1) that cause a 50% reduction in the viability of the MDCK cells. In this case, the IC_50_ value for the *C. mimosoides* extract is remarkably lower than the CC_50_ value, implying its effective antiviral concentration without inducing harmful effects on the host cells.

We assessed the antiviral activity of the extract by testing it against an influenza A H1N1 virus. Cytotoxicity assays against MDCK cells revealed a cytotoxicity concentration (CC_50_) greater than 100 µg/mL and identified a range of extract concentrations that proved non-toxic. Extracts were diluted within this range and pre-mixed with the virus prior to addition to MDCK cells for infection. The virus-compound mixture was left on the cells for 24 h, enabling assessment of antiviral activity throughout the course of virus infection and replication. Results showed that our plant extract was capable of inhibiting virus infection with an IC_50_ of 5.14 µg/mL (Figure 9B). Given that the compound was present from the attachment through replication and budding stages, inhibition may have occurred at any stage of the viral life cycle. The difference between IC_50_ and CC_50_ values demonstrates that the extract can be used at an effective antiviral concentration without detrimental effects on the host cells. As GA (1) is the main component of the extract, pure GA was also tested and found to inhibit H1N1 infection with an IC_50_ of 4.35 µg/mL and a CC_50_ higher than 100 µg/mL (Figure 9B,C).

While *C. mimosoides* is consumed regularly and has long been used as traditional medicine, its aqueous extract was tested with a hemolytic assay to ensure low toxicity toward human red blood cells (hRBCs), which can be considered a surrogate for normal cells. As shown in Figure 9A, even at the highest concentration tested (800 µg/mL), the plant extract did not show hemolytic activity, unlike the positive control Triton X-100. This indicates the potential applicability of *C. mimosoides* water extracts as an alternative and safe antiviral agent.

### 3.7. Molecular Docking for Anti-Influenza Activity

#### 3.7.1. Target Specificity towards Viral Surface Proteins

Molecular docking was implemented to explain mechanisms underlying the antiviral activity of the *C. mimosoides* aqueous extract. As mentioned, hemagglutinin (HA) and neuraminidase (NA) play a significant role in the process of viral entry and release of influenza viruses [12,13,14,15,16,17,60]. They have thus been promising candidates for developing targeted inhibitors to block critical steps of the viral cycle [60]. Molecular docking studies revealed that all of the complexities were predicted to have negative Gibbs free energies (ranging from −3.2 kcal/mol to −7.2 kcal/mol), indicating the reactions can proceed and are in accordance with the second law of thermodynamics (Table 4). The results showed that the target ligands preferentially interact with neuraminidase (NA: 6HP0) over hemagglutinin (HA: 1RU7), indicating that the former protein (NA) is the feasible target inhibitor for all metabolites detected in this study and should be further explored into the details to unravel a plausible mechanism of actions contributing the antiviral activity of the *C. mimosoides* aqueous extract.

#### 3.7.2. Molecular Docking of GA against NA

**Figure 10:**
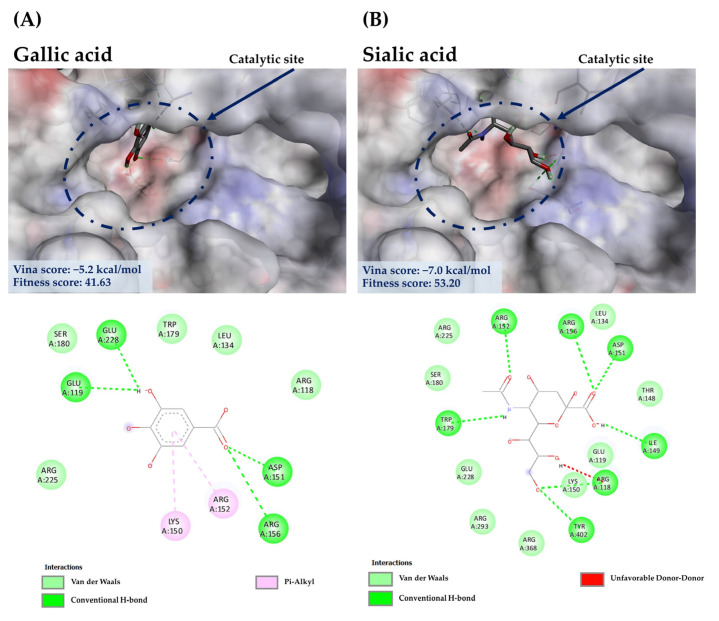
Molecular docking illustrating the localization of *C. mimosoides*’s metabolites in the two important regions (1 and 2) of NA; (**A**) gallic acid; and (**B**) sialic acid.

GA (1), a major component in the *C. mimosoides* aqueous extract, was first chosen to see whether it could interact with the important amino acid residues located in the active region of the NA structure, supporting the wet-lab experiments. This compound had a binding score of −5.2 kcal/mol, showing conventional hydrogen bond interactions with Glu119, Asp151 Arg156, and Glu228 along with forming π-alkyl interaction with Arg152 and Lys150, which is also encompassed by Arg118, Leu134, Trp179, Ser180, and Arg225 of the catalytic inner and outer shells of the active region. This result could be experimentally supported by the fact that the standard GA exhibits the IC_50_ of 4.53 µg/mL (Figure 9). It must also be stressed that GA also shares a similar binding to anti-neuraminidase agents, whose structures located in the catalytic region, including laninamivir, peramivir, oseltamivir, DANA, and zanamivir, possessing minimal binding energies of −5.4 kcal/mol, −6.6 kcal/mol, −6.5 kcal/mol, −6.2 kcal/mol, and −7.1 kcal/mol, respectively. Despite the difference between GA (1) and sialic acid in terms of binding, they share a similar conventional bonding formation towards the highly conserved residues in the defined region (Figure 10), including Arg118, Ile149, Asp151, Arg152, Arg156, Trp179, and Try401, is known to prevent viral infection and spreading [62]. Therefore, GA (1), a main phytochemical constituent, presumably plays a decisive role in disturbing the native function of NA. Further investigations (e.g., anti-NA activity assays) are needed to support this potential mechanism of action. To aid our prediction regarding the synergistic effects of other bioactive components, the GA’s binding property may also reflect the binding potential of other constituents, where those showing a free energy of more than −5.2 kcal/mol used for the comparison to explain the potential anti-neuraminidase activity, by considering their ability to interact with the highly conserved amino acid residues of the catalytic site and/or another region (i.e., 430-cavity). Meanwhile, metabolites having free binding energy below −5.4 kcal/mol will be considered behaviorally active, indicating a greater potential for binding.

#### 3.7.3. Molecular Docking of the Minor Constituents against NA Catalytic Region

**Figure 11:**
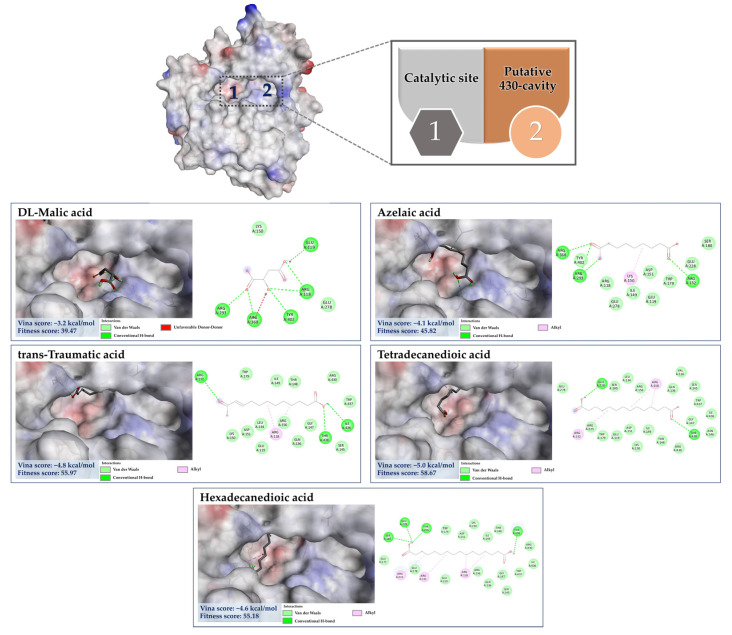
Illustrates a theoretical interaction between dicarboxylic acids of varying chain lengths and the catalytic site (1) within the NA structure.

Numerous studies have suggested that the enhanced efficacy of plant extracts is most likely a result of the synergistic effect of their major and minor constituents [63,64,65]. As detailed by CANOPUS, phenolics, and methylated analogs are biosynthetically derived from shikimate and phenylpropanoid pathways, suggesting that they feasibly share some structural, physicochemical, and biological properties. Taking into consideration the dicarboxylic acids (malic acid, azelaic acid, *trans*-Traumatic acid, tetradecanedioic acid, and hexadecanedioic acid) (Figure 11), whose free binding energies in the range from −3.2 kcal/mol to −5.2 kcal/mol. When taking a look closer at the binding profile, they can form a number of hydrogen bonds and hydrophobic interactions, signifying feasibility and strength of interactions and allowing for precise recognition between molecules towards the amino acid residues of active region and the 430-cavity of the NA structure. Among them, malic acid possessed the highest binding energy of −3.2 kcal/mol, showing H-bonding interaction with Arg118, Glu119, Arg293, Arg368, and Tyr402, which was similar to anti-neuraminidase drugs (e.g., laninamivir, peramivir, and oseltamivir), indicating its binding potential for NA structure. This could be partly supported by the study conducted by Rhee et al. [66], who demonstrated the virucidal efficacy of malic acid as one of seven active substances in commercial disinfectants against the H9N2 low pathogenic avian influenza virus. Thus, other longer-chain dicarboxylic acids, whose binding free energies are below −3.2 kcal/mol, were also presumably involved in the inhibitory effect against the influenza virus, as they form a substantial number of hydrogen bonds and alkyl-alkyl interactions with the amino acid residues of this region.

**Figure 12:**
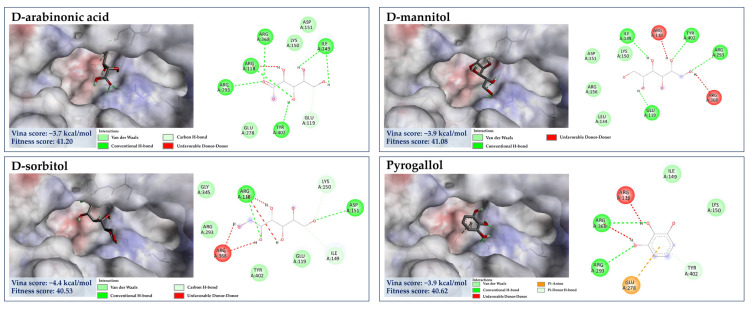
Hypothetical interactions of D-arabinonic acid, D-mannitol, D-sorbitol, and pyrogallol with highly conserved residues of the NA catalytic site.

Likewise, D-sorbitol, D-mannitol, D-arabinonic acid, and pyrogallol (a putative decarboxylated form of GA) presumably played a role in the antiviral activity due to the binding interactions with the catalytic site and lower binding energies when compared to that of malic acid, ranging −3.7 to −4.4 kcal/mol. D-sorbitol, for instance, had a binding score of −4.4 kcal/mol, showing H-bind interactions with Arg118, Asp151, Ile149, and Lys150 along with Vander walls forces with Glu119, Arg293, Tyr402, and Gly345 of the active pocket. Our result may confirm the recent finding that sorbitol exhibits mild activity against influenza H1N1 virus [67]. The direct evidence illustrating the anti-influenza activities of pyrogallol, D-arabinonic acid, and D-mannitol remains undocumented so far. These compounds, however, can generate a remarkable number of hydrogen bonds/π-anion interactions within the catalytic inner shell. For example, D-arabinonic acid (∆G = −3.7 kcal/mol) can form hydrogen binding with Arg118, Arg293, Arg368, and Tyr402, along with a π-anion interaction with Arg118 of the catalytic inner shell, which was similar to pyrogallol (∆G = −3.9 kcal/mol) and D-mannitol (∆G = −3.9 kcal/mol), signifying their promising antiviral activity towards NA’s active region (Figure 12). Remarkably, D-mannitol serves as a starting material in the asymmetric synthesis of oseltamivir [68], supporting the probability of our supposition.

**Figure 13:**
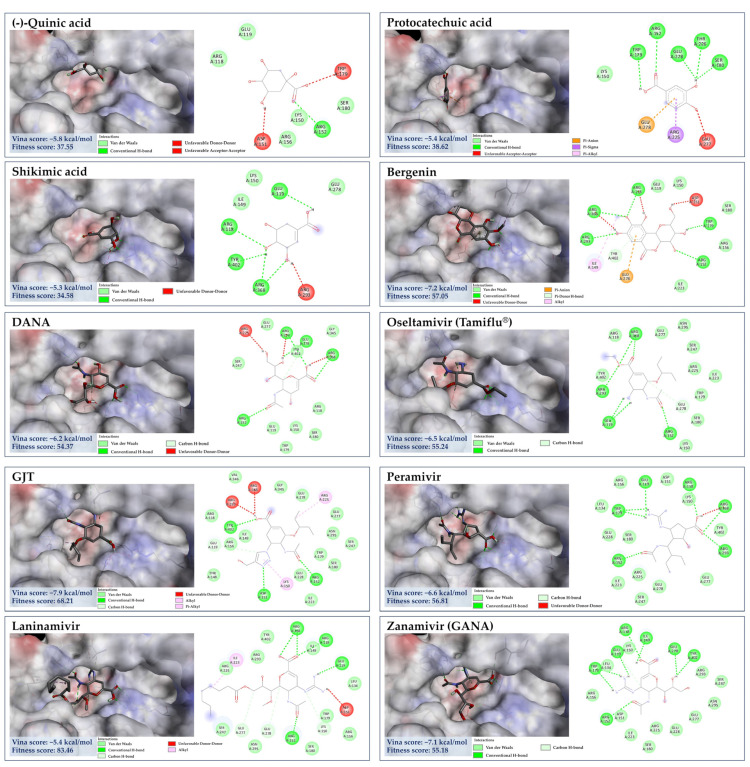
The hypothetical interactions of (-)-Quinic acid, protocatechuic acid, shikimic acid, and bergenin against the catalytic region of NA. DANA, oseltamivir, GJT, peramivir, laninamivir, and zanamivir served as the positive ligands located in this active region.

Aside from gallic acid, the top four binders within the NA catalytic site included quinic acid, shikimic acid, bergenin, and protocatechuic acid—are the second, fourth, fifth, and sixteenth most abundant phytochemicals present in the *C. mimosoides* aqueous extract. They exhibit strong interactions with amino acid residues that are highly conserved, akin to the anti-neuraminidase drugs, representing low binding energies of −5.8 kcal/mol, −5.3 kcal/mol, −7.2 kcal/mol, and −5.4 kcal/mol, respectively. Among them, bergenin is able to form the highest conventional and π-hydrogen bonds with Arg118, Arg152, Trp179, Arg 293, Arg368, and Tyr402, along with a π-anion interaction with Glu278 of the catalytic inner and outer shells, highlighting its promising anti-influenza activity although this assumption has not yet been experimentally elucidated, so far. Also, the protocatechuic acid having H-bonding interaction with Arg152, Trp179, Ser180, Thr226, and Glu228 along with π-anion, π-sigma, and π-alkyl interactions with Glu278 and Arg225 of the defined pocket (Figure 13). This evidence gains affirmation from Wang et al. [41], who demonstrated that it is able to protect the mice model from influenza A virus infection. Quinic acid and shikimic acid were also determined to show H-bonding interaction with Arg152, Arg118, Glu119, Arg368, and Tyr402, as it is always observed GJT, laninamivir, peramivir, oseltamivir, DANA, and zanamivir, thus supporting both phenolic substances play a pharmaceutical role in the synthetic routes of these anti-neuraminidase agents [56,69,70].

#### 3.7.4. Molecular Docking of the Minor Constituents against the 430-Cavity

**Figure 14:**
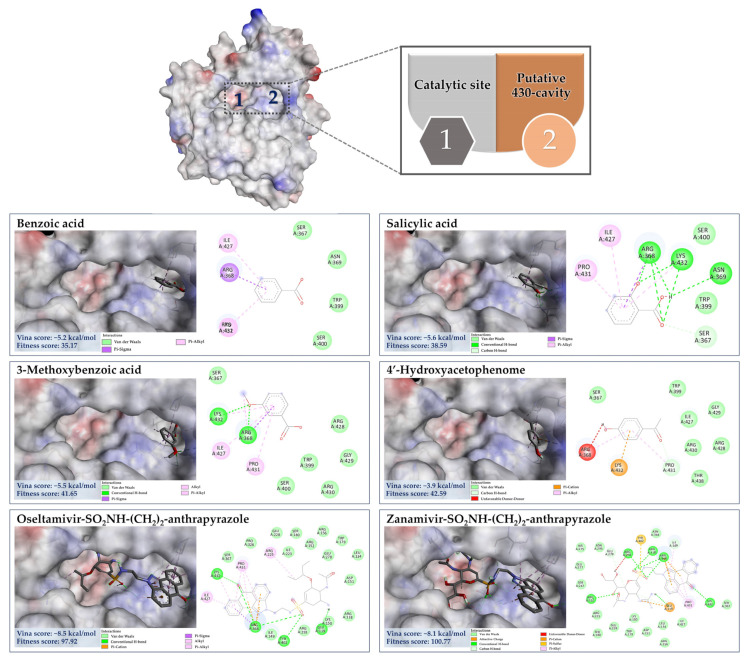
Molecular docking of the minor constituents is predicted to interact with the 430-cavity of the NA structure. Oseltamivir and zanamivir sulfamide-anthrapyrazole derivatives were chosen as reference ligands for this hydrophobic region [61].

X-ray crystallographic and NMR studies have confirmed that the NA also contains another region, the so-called “430-hydrophobic cavity”, characterized by Ser367, Ser370, Ser372, Ile427, Pro431, Asn400, Trp403, and Lys432 [62,71]. This region is, therefore, believed to play an essential role in engaging and recruiting multivalent sialosides to get closer to the catalytic inner shell and serve as the next generation in designing the new anti-influenza agents. Here, benzoic acid, salicylic acid, 3-Methylbenzoic acid, and 4′-Hydroxyacetophenone showed binding interaction with the putative 430-region by having the binding energies of −5.2 kcal/mol, −5.6 kcal/mol, −5.5 kcal/mol, and −3.9 kcal/mol, respectively. For example, 3-Methylbenzoic acid can form the conventional hydrogen bond with Lys432 and Arg368 (catalytic inner shell) with a number of hydrophobic interactions, including alkyl-alkyl interaction (Arg368), π-sigma (Arg368), and π-alkyl (Ile425, Pro431, and Lys432) of that pocket (Figure 14). 4′-Hydroxyacetophenone, predicted to receive the highest binding energy among the group (∆G = −3.9 kcal/mol), can also create a carbon–hydrogen bond with Pro431, π-cation (Lys432) coupled with π-alkyl interaction with Arg368 and Pro431 in this hydrophobic region. A similar interaction pattern was also observed for their parent structures—benzoic acid and salicylic acid. It is important to note that our ligands exhibit comparable binding interactions to reference ligands—oseltamivir and zanamivir sulfamide-anthrapyrazole derivatives (Table 5). They were designed to engage both the catalytic and 430-hydrophobic sites, addressing drug resistance in flu viruses. Markedly, the sulfamide-bridged anthrapyrazole components efficiently interact with the 430 region and key catalytic residues such as Ser367, Ile427, Pro431, Lys432, and Arg368 within the NA structure [61]. Our findings may also support the existing knowledge that hydrophobic benzoic acids inhibit the NA activity of influenza viruses, even those resistant to oseltamivir [72,73].

## 4. Discussion

Several studies have illustrated the applicability of plant aqueous extracts as a promising source of antiviral agents because of their large number of phenolic substances, such as hydroxybenzoic acids, hydroxycinnamic acids, and phenylpropanoids, whose biosynthetic route originate from the shikimate pathway [11,12,13,14,15]. In our previous work, we hypothesized that *C. mimosoides* could be a promising source of phenolic substances (e.g., GA (1), ferulic acid, vanillic acid, and resveratrol), but organic solvent extraction is costly and harmful to the environment [18,19,20]. Upon observation, the yield of GA (1) present in the *C. mimosoides* ethanolic extract is a significant decrease compared to that found in the aqueous extract (approx. 8.03-fold) (Figure 3). The high-resolution UPLC-ESI(+)-QTOF-MS/MS analysis also confirms that the *C. mimosoides* ethanolic extract contains a substantially lower concentration of GA (1) and other phenolic substances. This confirms the superior efficiency and cost-effectiveness of water-based extraction. Hence, we assessed the composition and activity of the aqueous extract of this plant to verify our suspicions and discovered a new, safe, and effective antiviral agent. In our study, the aqueous extract of *C. mimosoides* was pale yellow in color, resembling the physical characteristics of authentic gallic acid. (https://pubchem.ncbi.nlm.nih.gov/compound/Gallic-Acid#section=Experimental-Properties, accessed on 1 August 2023). The antiviral properties of the aqueous extract are directly related to the total phenolic content (TPC) and radical scavenging activities [5,6,7,8]. Consequently, this aqueous extract has a promising TPC concentration and potent anti-oxidant activity, with a slightly higher IC_50_ value than GA (1). This indicates our success in preparing the plant water extract with a significant number of antioxidating agents, aligning with our initial objective. Based on our previous research, we postulated that GA (1) is also the primary constituent in the aqueous extract of *C. mimosoides*.

HPLC analysis was performed to validate this hypothesis. The putative peak associated with GA (1) was found to be a majority in the plant water extract, at 93.27% of the total amount of metabolites detected. Therefore, this compound is moderately soluble in water, which indicates that it has powerful antioxidant properties (https://pubchem.ncbi.nlm.nih.gov/compound/Gallic-Acid#section=Solubility, accessed on 1 August 2023). Our results are also consistent with those of Singh et al. [74], who demonstrated the efficiency of water over ethanol in extracting GA (1) from pomegranate aril. However, the data were insufficient to provide structural details for the remaining metabolites (around 6.73%) in the aqueous extract, in addition to the probable GA (1). To address this issue, untargeted screening analyses using the UPLC-ESI-QTOF-MS/MS in negative ion mode, which is known to be very sensitive and appropriate for assessing phenolics and compounds containing hydroxyl [53,54,55], were implemented. As a result, the GA (1) (*m*/*z* 169.0217; [M-H]^−^) had the highest relative mass intensity (3.396 × 10^7^) among the other metabolites and had emerged in the negative ion mode. Therefore, we were fairly confident that it was the main phenolic present in the water extract of *C. mimosoides*.

At a lower relative peak intensity (7.78 × 10^6^), the putative pyrogallol (*m*/*z* 125.0258, [M-H]^−^, Rt = 2.93 min) was detected as a co-eluting product of GA (1), indicating both metabolites have similar characteristics in terms of physicochemical properties and biosynthetic origin (shikimic acid pathway) [75], since this minor metabolite has been proposed as the enzymatic product of GA (1) or 2,3-dihydroxybenzoic acid (2,3-DHBA) catalyzed by the genes encoding for PCA decarboxylase (aroY), 3,4-dihydroxybenzoic acid decarboxylase (PDC), and 2,3-DHBA 1-monooxygenase [56]. This phenomenon also led us to believe that the main peak of GA (1) (93.27% peak area) observed in the HPLC analysis (Figure 3) could also be detected by conventional UV spectroscopy. Further elucidation, e.g., optimization of HPLC conditions, must be supported from a chemical point of view.

Furthermore, various metabolites tentatively classified as phenolic acids, methylated products, sugar derivatives, and dicarboxylic acids were also present in the same aqueous extract with values almost identical to those compounds in the in-house MS library. When compared to the literature, the first two groups (phenol and its methylated derivatives) appear to originate from the shikimic and phenylpropanoid pathways with a common aromatic core structure (i.e., benzoic acid) but differ only in some structural modifications, such as methylation, glycosylation, decarboxylation, dehydration and hydroxylation [76,77,78], indicating that the annotation of native MS libraries is quite reliable. For instance, bergenin is a product derived from glycosylation and methylation of GA (1), while pyrogallol acts as a decarboxylated form of GA (1) [56,76,77,78]. However, they still completely lack information on correct molecular formulas, candidate structures, molecular fingerprints, and classifications, which need to be addressed using mass spectrometry annotation tools. After careful consideration, all four classes of natural products were subjected to structure-assisted annotation by a series of software, including the MetFrag web service, SIRIUS, CSI:FingerID, and CANOPUS, to meet the results obtained according to the metabolomics initiative guidelines [37,38,39,40].

MetFrag is an in silico tool designed to directly convert raw MS/MS spectra of metabolites of interest by matching them with in silico fragment MS/MS spectra of substances retrieved from databases (e.g., PubChem, NORMAN, and KEGG) [40]. After submitting the raw data, 6 out of 24 metabolites, including the negative precursor ions at *m*/*z* 369.0684, 193.0371, 165.0926, 455.2501, 315.2536, and 166.8340, could not be identified using these tools despite consistent values (84.7% to 100% similarity) and hypothesized potential structures from an in-house MS library (Table 3). This occurs for two main reasons: (1) the signal strength of the query metabolite is not sufficient for the annotation experiment, or (2) the collision-induced dissociation energy (CID) is not sufficient to produce a unique fragmentation pattern of the target metabolite, resulting in unsuccessful query compound and match set between reference compounds [79]. To see the details, consider the precursor ions of *m*/*z* 166.8340 [M-H]^−^ and 455.2501 [M-H]^−^ as examples. The local MS library had an identical value of 88.1%, indicating that the previous ion was most likely isorhamnetin, but only one of the three precursor ions (*m*/*z* 315.2534) matched the database. Although the correct chemical formula (C_16_H_12_O_7_) has been reported, this result is currently unreliable because MetFrag cannot account for any mass peak of the putative isorhamnetin (0/1: final result = 0). Advanced search capabilities were based on the Plant Metabolome Database (PDMB), as well as azaleatin (5-*O*-Methylquercetin), which is an isomeric methylated analog of isorhamnetin and has the same elemental formula (https://scbt.sastra.edu/pmdb/eadvance.php, accessed on 4 August 2023). Therefore, it is reasonable to exclude “isorhamnetin” as a candidate for *m*/*z* 315.2534 [M-H]^−^, since other methylated analogs may exist in plant cells, such as rhamnetin, tamarixetin and azaleatin [80,81,82]. Likewise, the putative 3-hydroxy-4-methoxybenzoic acid (*m*/*z* 166.8340 [M-H]^−^) had a library score of 84.7% and could not be further annotated using MetFrag because no product ions were consistent with those in the database ions. This suggests that matching only one pair of precursor ions is not sufficient to provide accurate details about the query subject. These six metabolites cannot then be evaluated coherently by a range of software to extract additional information from large MS/MS data sets. Despite this being the case, MetFrag indicated that the remaining 18 metabolites might have annotations that match library-matching events. Most of them could be assigned to the first candidate (1st place), while some of them were assigned to different rankings (such as 2nd, 5th, 7th, and 20th). Therefore, further explanation is needed to increase the likelihood of its accuracy. Although NMR spectroscopy is necessary to verify the presence of trace components in the aqueous extract of *C. mimosoides*, this strategy was considered unfeasible in our case due to its low sensitivity, implying the need for alternative strategies.

Our recent study, using SIRIUS combined with CSI:FingerID and CANOPUS, demonstrated efficient and rapid structure elucidation of 2,4,6-trihydroxybenzophenone and ferulenol produced by engineered *E. coli* [83,84]. From the same perspective, the correct identification rate for 17 phytochemicals (except 3-hydroxy-4-methoxy cinnamic acid) was significantly higher compared to the top candidate (1st), indicating that the results were more reliable. According to Dührkop et al. [38], CSI:FingerID integrated into SIRIUS is extremely effective for identifying query subjects when MS/MS data from independent sources is included in the training dataset; disjointing those data results in a 27.6% decrease in identification confidence [38]. When compared to those previously annotated by the MetFrag web service, the newly defined rank as “the first candidate” for hexadecanedioic acid, shikimic acid, 1,2,3-benzenetriol, GA (1), protocatechuic acid, benzoic acid, and 3-methoxybenzoic acid supports this approach. The class annotations, for example, for phenolics and methylated derivatives, also extend those of Wu et al. [56], confirming that these molecules have extremely close chemical relationships in terms of their molecular structures. In contrast, other candidates (isomeric structures) ranked 2nd–100th in the CSI:FingerID analyses may be disqualified as being the wrong structures for the query subjects. It is important to note that other isomeric structures are still viable candidates because the structural databases are still lacking natural product diversity and are incomplete [56]. Most importantly, the candidate structures for the 17 metabolites predicted by SIRIUS should be noticeably reliable because the accuracy of these results could be verified by an InChI search using the CSI:FingerID web service’s negative mode data. (accessed on 20 July 2023 at https://www.csi-fingerid.uni-jena.de/v2.6/api/fingerid/trainingstructures?predictor=1). Our findings offer strong support for the structural annotation of natural products using silico-based methods. This technique appears to be very effective and reliable in extracting key information (such as the molecular formula, structure, and classes) from experimental MS^2^ data of natural products. Future research should examine whether these cheminformatics tools are retained over time so that they can be used to identify phenolic compounds in other plant extracts (e.g., NMR and standard comparison).

Our aqueous plant extract, which was tested against the influenza A/Puerto Rico/8/34 (H1N1) virus to see if it could be repurposed as an antiviral agent as anticipated, was known to be a safe and rich source of antioxidant substances. The extract’s impressive IC_50_ value (4.35 µg/mL) was shown to be able to efficiently inhibit this virus strain, extending earlier findings that aqueous extracts containing a number of phenylpropanoid-derived compounds (e.g., GA (1), vanillic acid, and resveratrol) are promising sources of natural anti-influenza agents targeting NA and HA [9,10,11,55,65,68,69,84]. Our results also corroborate the existing knowledge, wherein the alcoholic extracts of *C. minax* seeds and *C. latisiliqua* twigs, abundant with flavonoids, stilbene, phenolics, and other compounds, demonstrate promising antiviral activity [30,31]. Since the two viral surface proteins play critical roles in viral infection and spread, molecular docking was used to understand the potential mechanisms underlying the antiviral activity of *C. mimosoides* extract [59]. However, the A/Puerto Rico/8/34 (H1N1) influenza A virus’s crystallographic NA structure is not available in the PDB database. The crystal NA structure (PDB ID: 6HP0; X-ray diffraction 1.88 Å) originating from the influenza strain (A/Texas/17/2009(H1N1), which is co-crystallized with the oseltamivir triazole derivative, was chosen instead due to the absence of mutation in the catalytic and substrate binding residues with 86% identity to the expected sequence. Molecular docking reveals that nearly all phytochemicals tend to be more specific towards the NA (PDB: 6HP0) than the HA (PDB: 1RU7) when looking for target specificity (Table 4). Thus, the NA protein was selected as the more promising target for the antiviral activity seen in this study.

The main component of the *C. mimosoides* water extract, GA (1), interacts specifically at the catalytic region (also known as the “inner shell”) of the NA, sharing the same pocket as sialic acid and other anti-neuraminidase medications commonly prescribed in hospitals, such as laninamivir, peramivir, oseltamivir, and zanamivir (DANA) [62]. This finding is similar to that of Zhang et al. [85], who showed that the GA (1) isolated from the ethanolic extract of *Radix Paeonie Alba* inhibits influenza (H1N1) by interacting with the Arg152 of the neuraminidase enzyme as well [85]. It also highlights the important role of GA (1) as a specific inhibitor of influenza virus NA. When taking into account the fundamental interaction between NA and sialic acid, the synergistic effects between GA (1) and other minor constituents may also be possible due to their specific affinity towards the highly conserved residues in the catalytic region of the viral surface enzyme. This possibility is further supported by the antiviral activities of protocatechuic acid, pyrogallol, quinic acid, malic acid, and D-sorbitol [41,65,68,69,84], with the skeleton role of shikimic acid in the synthetic routes of oseltamivir (Tamiflu^®^) and other derivatives, i.e., GJT (PubChem CID: 139030257) [69,70,86]. In addition, NA contains a second site that, under some conditions, is thought to act like the HA protein by bringing multivalent sialosides closer to the catalytic region [87,88]. The hydrophobic surface created in the additional site by the interaction of the 430-cavity (herein detected as Lys432) and highly conserved residues (Pro326, Ile427, and Thr439) has been a target in the design of enzyme inhibitors with lipophilic portions [62]. By relying primarily on interactions between their lipophilic moieties (such as the methoxy group and aromatic ring system) and the hydrophobic cluster of that region, molecular modeling has shown that benzoic acid, salicylic acid, and three methylated derivatives can occupy the hydrophobic surface. We therefore proposed that the physicochemical characteristics, which can affect how well-suited metabolites succeed in localizing to the particular region of the NA framework, are likely to determine target site selectivity. These results might support the current knowledge that hydrophobic benzoic acids can inhibit the NA activity of influenza viruses, even those that are resistant to oseltamivir, as indicated in studies by Atigadda et al. [72] and Guo et al. [73]. Our hypothesis may be confirmed by Evteev et al. [61], who found that the hydrophobic 430-cavity of influenza NA may interact with the polycyclic ring system of the anthrapyrazole fragment used in anti-NA medications (oseltamivir/zanamivir). Based on these results, it is likely that benzoic acid and its four related derivatives act as second-site inhibitors, which could account for how they work in concert with phytochemicals caged in the inner shell (such as GA (1), hexadecanedioic acid, and sorbitol) by keeping multivalent sialosides away from the NA catalytic region. This would enable the effective confinement of viral progeny and their dissemination to new target cells, where the stability issues need to be further consolidated using in vitro experiments (e.g., isothermal titration calorimetry) and in silico-based analyses such as molecular dynamic simulation. The literature claims that a higher mutation rate near the NA active region has caused a gradual decline in anti-influenza drug effectiveness, necessitating the search for new promising sources of bioactive agents that could simultaneously interact with other controlling sites (i.e., the 430-cavity) to disrupt enzyme activity [61,62]. The antiviral activity of the *C. mimosoides* aqueous extract against oseltamivir-resistant influenza A (H1N1), and the possible synergy between the phytochemicals present are being.

## 5. Conclusions

We demonstrated that the aerial parts of *C. mimosoides*, can be considered a promising source of bioactive constituents, as evidenced by the potent antioxidant and good anti-influenza (H1N1) activities of its water extract, where GA (1) served as the main constituent based upon the HPLC analyses. Apart from the absence of hemolytic properties, numerous bioactive substances were also deduced via the use of untargeted metabolomics analyses (LC-MS/MS) for the first time, where the various cheminformatics tools supported high-confidence structural descriptions of those metabolites. Docking studies predicted that the NA protein was the main target of those annotated phytochemicals, which could interact with the NA catalytic site and the hydrophobic 430-cavity, implying a potential synergistic effect. Molecular dynamic simulation and isothermal titration calorimetry are required to see the impacts of environmental factors (e.g., pH, ionic strength, and temperature) on the receptor-ligand complexes, addressing and consolidating the obtained results. This aqueous extract may be a promising choice for dealing with oseltamivir-resistant strains, as there is an urgent need for bioactive agents that can simultaneously interact with the two crucial sites of the NA structure. Phenolic compounds have been constantly evidenced to demonstrate a remarkable capacity to target multiple pathways, illuminating the potential of *C. mimosoides* aqueous extract as a versatile therapeutic agent capable of addressing complex diseases with diverse origins.

## Figures and Tables

**Table 1:** Total phenolic content of *C. mimosoides* aqueous extract.

Fresh Aerial *C. mimosoides* Parts (g)	Dried Material (g)	Total Phenolic Content (g)
15.00	3.11	1.71
		551 mg (GAE)/g. dried weight

**Table 2:** Structural information of selected metabolites of plant aqueous extract based on matching with the in-house LC-MS/MS library.

No.	Exp.RT/RT (Minute)	Predicted Metabolite	Neutral Formula	Neutral Mass (*m*/*z*)	Precursor Mass (*m*/*z*) [M-H]^−^	Exp. Mass (*m*/*z*) [M-H]^−^	Mass Error (ppm)	Library Score (%)	Relative Peak Area	Relative Peak Area ^1^ (*%*) (All Metabolites)/Rank	Relative Peak Area ^2^ (*%*) (24 Metabolites Detected)/Rank	Relative Peak Area ^3^ (*%*) (18 Metabolites Selected)/Rank
1	0.71/0.80	DL-Malic acid	C_4_H_6_O_5_	133.87470	132.868	132.8682	1.505253	100	6.026 × 10^5^	0.58/**21**	0.82/**10**	0.87/**7**
2	0.71/0.88	Salicylic acid	C_7_H_6_O_3_	137.86958	136.863	136.8630	0	100	1.966 × 10^5^	0.19/**43**	0.27/**16**	0.28/**11**
3	0.71/0.75	Digalactouronic acid	C_12_H_18_O_13_	370.07154	369.068	369.0684	1.083811	95.2	1.049 × 10^5^	0.10/**54**	0.14/**23**	-
4	0.75/0.77	D-arabinonic acid	C_5_H_10_O_6_	166.04819	165.041	165.0414	2.423640	96.8	1.690 × 10^5^	0.16/**47**	0.23/**19**	0.24/**14**
5	0.75/0.75	D-sorbitol	C_6_H_14_O_6_	182.07596	181.073	181.073	0	95.7	2.466 × 10^5^	0.24/**39**	0.34/**14**	0.35/**10**
6	0.75/0.76	5-keto-D-gluconic acid	C_6_H_10_O_7_	194.04362	193.037	193.0371	0.518000	95.5	8.989 × 10^5^	0.87/**14**	1.22/**7**	-
7	0.87/0.88	(-)-Quinic acid	C_7_H_12_O_6_	192.06664	191.060	191.0607	3.663771	98.7	1.301 × 10^7^	12.53/**2**	17.73/**2**	18.72/**2**
8	1.13/1.14	Shikimic acid	C_7_H_10_O_5_	174.05405	173.047	173.0472	1.155755	99.4	3.766 × 10^6^	3.63/**5**	5.13/**4**	5.42/**4**
9	2.92/2.93	Pyrogallol	C_6_H_6_O_3_	126.03248	125.026	125.0258	−1.599667	98.9	7.780 × 10^6^	7.49/**4**	10.60/**3**	11.20/**3**
10	2.92/2.93	Gallic acid	C_7_H_6_O_5_	170.02842	169.022	169.0241	12.424418	96.3	3.967 × 10^7^	38.21/**1**	54.06/**1**	57.09/**1**
11	3.53/3.51	Protocatechuic acid	C_7_H_6_O_4_	154.02666	153.020	153.020	0	97.6	1.146 × 10^5^	0.11/**50**	0.16/**21**	0.16/**16**
12	3.69/3.70	Bergenin	C_14_H_16_O_9_	328.08096	327.074	327.0743	0.917224	96.7	1.410 × 10^6^	1.36/**9**	1.92/**5**	2.03/**5**
13	4.13/4.09	4′-hydroxyacetophenone	C_8_H_8_O_2_	136.08886	135.082	135.0822	1.480582	83.3	7.230 × 10^5^	0.70/**17**	0.99/**9**	1.04/**6**
14	4.13/4.06	1,2-Benzenedicarboxylic acid	C_13_H_16_O_4_	166.09928	165.093	165.0926	−2.422877	99.2	5.482 × 10^5^	0.53/**25**	0.75/**13**	-
15	4.13/4.08	Benzoic acid	C_7_H_6_O_2_	122.07921	121.066	121.0663	0.247920	62.4	5.680 × 10^5^	0.55/**23**	0.77/**11**	0.82/**8**
16	4.29/4.30	Azelaic acid	C_9_H_16_O_4_	188.10488	187.098	187.0982	1.068959	85.2	1.737 × 10^5^	0.17/**45**	0.24/**17**	0.25/**12**
17	4.33/4.31	Betulinuc acid	C_30_H_48_O_3_	456.25676	455.250	455.2501	0.219660	100	8.733 × 10^5^	0.84/**15**	1.19/**8**	-
18	4.40/4.39	3-Methoxybenzoic acid	C_8_H_8_O_3_	152.04700	151.040	151.0402	1.324153	93.2	6.116 × 10^4^	0.06/**58**	0.08/**24**	0.09/**18**
19	4.45/4.58	*trans*-Traumatic acid	C_12_H_20_O_4_	228.20954	227.203	227.2021	−3.961215	88.5	1.643 × 10^5^	0.16/**49**	0.22/**20**	0.24/**15**
20	4.56/4.64	3-Hydroxy-4-methoxycinnamic acid	C_10_H_10_O_4_	194.09463	193.088	193.0878	−1.035797	72.3	1.051 × 10^5^	0.10/**53**	0.14/**22**	0.15/**17**
21	4.76/4.73	Hexadecanedioic acid	C_16_H_30_O_4_	286.21477	285.208	285.2081	0.350621	99.7	1.708 × 10^5^	0.16/**46**	0.23/**18**	0.25/**13**
22	4.84/4.84	Isorhamatin	C_16_H_12_O_7_	316.26016	315.253	315.2536	1.903233	88.1	1.233 × 10^6^	1.19/**10**	1.68/**6**	-
23	4.84/4.85	Tetradecanedioic acid	C_14_H_26_O_4_	258.18427	257.178	257.1776	−1.555343	95.9	5.536 × 10^5^	0.53/**24**	0.75/**12**	0.80/**9**
24	5.84/5.89	3-hydroxy-4-methoxybenzoic acid	C_8_H_8_O_4_	167.84063	166.834	166.8340	0	84.7	2.432 × 10^5^	0.23/**40**	0.33/**15**	-

^1^ Calculated from entire metabolites present in the sample plant extract. ^2^ Calculated from 24 metabolites possessed the library score reading 62.4–100%. ^3^ Based on information annotated by MetFrag.

**Table 3:** Structural annotation of twenty-four metabolites based upon MetFrag.

No.	Retention Time (Minute)	Putative Peak * (*m*/*z*; [M-H]^−^)	Neutral Formula	Database (Rank/Entire Candidate; F1 Score) Monitoring Based on In-House Library				(Number of Peaks) Matched Peaks ● Matched ● Not matched ● Excluded	Candidate Structure ** (InChIKeyBlock1)
				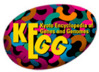	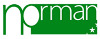	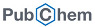	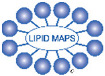		
1	0.71	DL-Malic acid (133.0137)	C_4_H_6_O_5_	(1/4: 1.0)	N.D.	(42/153: 0.8173)	N.D.	(4/4) 71.0144 72.9938 89.0256 115.0044 132.8683 133.0137	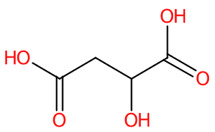 BJEPYKJPYRNKOW
2	0.71	Salicylic acid (136.8630)	C_7_H_6_O_3_	N.D.	(1/13: 0.8173)	(31/308: 0.6744)	N.A.	(2/2) 65.0402 93.0353 136.8630	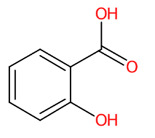 YGSDEFSMJLZEOE
3	0.71	Digalactouronic (369.0684)	N.D.	N.D.	N.D.	N.D.	N.A.	N.D.	N.D.
4	0.75	D-arabinonic acid (165.0414)	C_5_H_10_O_6_	(1/6: 1.0)	(1/1: 1.0)	(11/112: 0.7562)	N.A.	(5/7) 59.0144 71.0150 72.9941 75.0092 78.9599 99.0097 129.0204 164.8369 165.0416	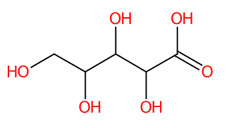 QXKAIJAYHKCRRA
5	0.75	D-sorbitol (181.073)	C_6_H_14_O_6_	(1/6: 1)	(1/1: 1.0)	(26/185: 0.7835)	N.A.	(4/13) 55.0193 57.0359 59.0142 71.0147 73.0303 78.9592 83.0137 85.0310 89.0251 93.0365 96.9613 101.0263 153.0316 180.8408 181.0726	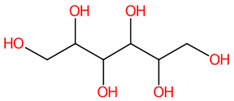 FBPFZTCFMRRESA
6	0.75	5-keto-D-gluconic acid (193.0371)	N.D.	N.D.	N.D.	N.D.	N.A.	N.D.	N.D.
7	0.87	(-)-Quinic acid (191.0607)	C_7_H_12_O_6_	(1/5: 1)	(2/4: 0.8985)	(126/702: 1.0)	N.A.	(4/7) 59.0145 85.0303 87.0095 93.0354 109.0304 111.0460 127.0407 191.0583	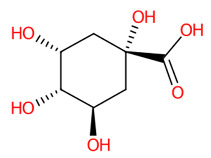 AAWZDTNXLSGCEK
8	1.13	Shikimic acid (173.0472)	C_7_H_10_O_5_	(5/5: 0.6902)	(4/10: 0.6902)	(193/170: 0.4491)	N.A.	(5/11) 55.0195 71.0147 73.0302 76.9705 81.0352 83.0509 93.0356 111.0459 136.9378 137.0252 154.9485 173.047	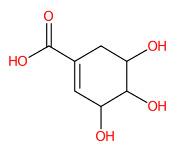 JXOHGGNKMLTUBP
9	2.92	Pyrogallol (125.0258)	C_6_H_6_O_3_	(5/7: 0.7328)	(7/12: 0.7328)	(216/365: 0.6497)	N.A.	(8/11) 51.0250 67.0197 69.0348 78.9680 79.0198 79.9592 81.0352 95.0139 96.9604 97.0299 107.0143 123.0092 124.0173 125.0246	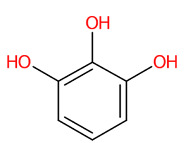 WQGWDDDVZFFDIG
10	2.92	Gallic acid (169.0241)	C_7_H_6_O_5_	(1/1: 1.0)	(2/2: 1.0)	(89/146: 0.6436)	N.A.	(4/7) 69.0354 79.0199 81.0353 97.0304 107.0147 124.0175 125.0269 169.0156	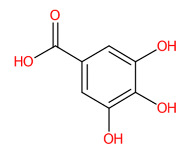 LNTHITQWFMADLM
11	3.53	Protocatechuic acid (153.0200)	C_7_H_6_O_4_	(2/4: 1.0)	(4/13: 1.0)	(16/325: 0.9806)	N.A.	(3/3) 91.01903 108.0222 109.0300 153.020	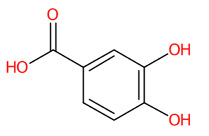 YQUVCSBJEUQKSH
12	3.69	Bergenin (327.0743)	C_14_H_16_O_9_	(1/2: 1.0)	N.D.	(2/89: 0.9733)	N.A.	(15/15) 164.0122 166.0280 178.0282 190.0284 192.0077 193.0154 194.0231 205.0156 206.0232 207.0312 222.0184 234.0184 237.0418 249.0418 312.0499 327.0732	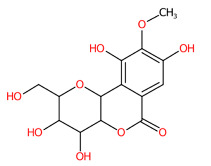 YWJXCIXBAKGUKZ
13	4.13	4-hydroxyacetophenone (135.0822)	C_8_H_8_O_2_	(1/15: 1.0)	(2/27: 1.0)	(21/833: 0.7430)	N.A.	(4/4) 92.0276 93.0353 108.0224 120.0225 134.8658 135.0462	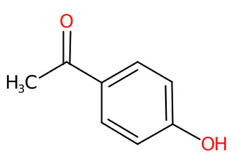 TXFPEBPIARQUIG
14	4.13	1,2-Benzenedicarboxylic acid (165.0926)	N.D.	N.D.	N.D.	N.D.	N.A.	N.D.	N.D.
15	4.13	Benzoic acid (121.0663)	C_7_H_6_O_2_	(5/6: 0.7078)	(9/9: 0.6598)	(216/298: 0.5669)	N.D.	(2/4) 77.0412 92.0265 93.0365 108.0212 119.0510 121.0308 121.0653	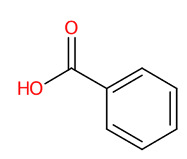 WPYMKLBDIGXBTP
16	4.29	Azelaic acid (187.0982)	C_9_H_16_O_4_	(1/2: 1)	(4/33: 0.8571)	(531/3149: 0.6948)	(1/1: 1.0)	(10/17) 57.03487 59.0141 71.0510 83.0507 85.0673 97.0664 99.0822 123.0828 125.0610 125.0979 141.1291 143.0517 143.1091 159.0646 168.8642 169.0876 169.1241 187.0983 187.1354	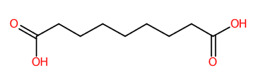 BDJRBEYXGGNYIS
17	4.33	Betulinuc acid (455.2502)	N.D.	N.D.	N.D.	N.D.	N.A.	N.D.	N.D.
18	4.40	3-Methoxybenzoic acid (151.0402)	C_8_H_8_O_3_	N.D.	(20/40: 0.7570)	(168/1077: 0.5668)	N.A.	(4/4) 92.0275 93.0353 108.0226 136.0176 151.0061 151.0413	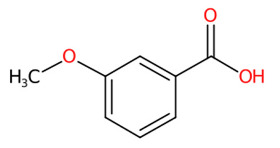 XHQZJYCNDZAGLW
19	4.45	*trans*-Traumatic acid (227.2021)	C_12_H_20_O_4_	(1/1: 1.0)	(1/15: 1.0)	(187/3696: 0.8825)	(2/5: 1.0)	(5/7) 57.03475 97.06573 126.9052 131.0720 139.1134 165.12972 183.13998 226.8016 227.0368 227.1295 227.1669 227.2030	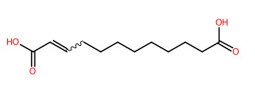 MAZWDMBCPDUFDJ
20	4.56	3-Hydroxy-4-methoxycinnamic acid (193.0878)	C_10_H_10_O_4_	(6/9: 1.0)	(15/32: 1.0)	(837/2148: 0.6964)	N.A.	(2/2) 177.0566 178.0643 193.0500	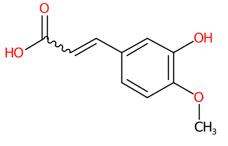 QURCVMIEKCOAJU
21	4.76	Hexadecanedioic acid (285.2081)	C_16_H_30_O_4_	(2/2: 1.0)	(2/15: 1.0)	(82/1425: 0.8668)	(2/3: 1.0)	(3/4) 59.0142 223.0293 223.2079 267.1981 285.1173 285.2086	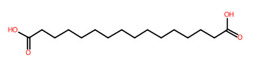 QQHJDPROMQRDLA
22	4.83	Isorhamatin (315.2536)	N.D.	N.D.	N.D.	N.D.	N.D.	N.D.	N.D.
23	4.83	Tetradecanedioic acid (257.1776)	C_14_H_26_O_4_	N.D.	(1/21: 1.0)	(91/2047: 0.7704)	(1/4: 1.0)	(4/6) 83.0509 183.0129 195.1765 211.2086 213.1880 239.1665 257.0506 257.1769 257.2139	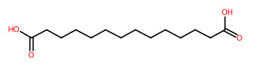 HQHCYKULIHKCEB
24	5.84	3-hydroxy-4-methoxybenzoic acid (166.8340)	N.D.	N.D.	N.D.	N.D.	N.D.	N.D.	N.D.

* Structural annotation was based on the default values of the software. ** The candidate structures were selected based on their correspondence with the LC-MS-based annotation. N.D.: Not detected. N.A.: Not applicable.

**Table 4:** Prediction of the target specificity of all ligands towards the two viral surface proteins.

No.	Compounds	PubChem CID	HA (PDB: 1RU7)		NA (PDB: 6HP0)	
			Fitness Score (GOLD)	Vina Score (CB Dock) (kcal/mol)	Fitness Score (GOLD)	Vina Score (CB Dock) (kcal/mol)
*C. mimosoides*’s metabolites						
1	Hexadecanedioic acid	10459	53.78	−3.9	55.18	−4.6
2	Tetradecanedioic acid	13185	56.72	−4.4	58.67	−5.0
3	Bergenin	66065	41.19	−5.7	57.05	−7.2
4	*trans*-Traumatic acid	5283028	50.48	−4.7	55.97	−4.8
5	Azelaic acid	2266	43.83	−4.2	45.82	−4.1
6	Gallic acid	370	36.34	−4.1	41.63	−5.2
7	D-arabinonic acid	122045	34.48	−3.7	41.20	−3.7
8	Pyrogallol	1057	41.38	−4.2	40.62	−3.9
9	D-sorbitol	5780	35.64	−3.3	40.53	−4.4
10	D-mannitol	6251	36.56	−3.8	41.08	−3.9
11	DL-Malic acid	525	31.69	−3.1	39.47	−3.2
12	Protocatechuic acid	72	37.05	−4.7	38.62	−5.4
13	(-)-Quinic acid	6508	28.74	−4.3	37.55	−5.8
14	Shikimic acid	8742	32.23	−4.2	34.58	−5.3
15	3-Methoxybenzoic acid	11461	43.83	−4.7	41.65	−5.5
16	4′-Hydroxyacetophenone	7469	36.64	−2.5	42.59	−3.9
17	Salicylic acid	338	43.55	−4.2	38.59	−5.6
18	Benzoic acid	243	36.16	−5.0	35.17	−5.2
Control ligand: Natural substrate						
1	Sialic acid	445063	42.37	−4.6	53.20	−7.0
Control ligand: anti-influenza agents						
1	GJT	139030257	42.59	−6.3	68.21	−7.9
2	Laninamivir	9847629	55.47	−5.1	83.46	−5.4
3	Peramivir	154234	40.49	−4.6	56.81	−6.6
4	Oseltamivir (Tamiflu^®^)	65028	48.14	−4.7	55.24	−6.5
5	DANA	65309	39.99	−4.9	54.37	−6.2
6	Zanamivir (GANA)	60855	44.35	−4.2	53.18	−7.1
7	Oseltamivir-SO2NH-(CH2)2-anthrapyrazole	Evteev et al. [61]	70.42	−5.8	97.92	−8.5
8	Zanamivir-SO2NH-(CH2)2-anthrapyrazole	Evteev et al. [61]	71.26	−6.3	100.77	−8.1

**Table 5:** The molecular interaction between target ligands and neuraminidase enzyme.

No.	Compound Name	Chemical Bond Interaction				
		H-Bond ● Conventional H-Bond ● Carbon H-Bond ● π-Donor H-Bond	Charge ● π-cation ● π-anion ● Attractive Charges	Hydrophobic ● Alkyl ● π-sigma ● π-alkyl ● π-sulfur	Van der Waals (VdW)	Unfavorable ● Acceptor-Acceptor Clashes ● Donor-Donor Clashes
*C. mimosoides*’s metabolites						
1	Hexadecanedioic acid	● Conventional H-Bond Ser180 (4.83 Å) Thr226 (4.19 Å) Glu228 (4.82 Å) Thr438 (4.31 Å)	N.D.	● Alkyl Arg118 (5.45, 5.48 Å) Arg152 (5.77 Å) Arg225 (6.94 Å)	● VdW Glu119, Gln136, Ser145, Gly147, Thr148, Ile149, Lys150, Asp151, Arg156, Trp179, Glu277, Glu278, Arg430, Ile436, Trp437	N.D.
2	Tetradecanedioic acid	● Conventional H-Bond Glu228 (4.95 Å) Thr438 (4.00 Å)	N.D.	● Alkyl Arg118 (4.47, 4.88 Å) Agr152 (6.32 Å)	● VdW Val116, Glu119, Leu134, Gln136, Ser145, Asn146, Gly147, Thr148, Lys150, Asp151, Arg156, Trp179, Ser180, Arg225, Glu278, Arg430, Ile436, Trp437	N.D.
3	Bergenin	● Conventional H-Bond Arg118 (6.48 Å) Arg152 (3.82 Å) Trp179 (4.62 Å) Arg293 (6.82 Å) Arg368 (6.32 Å) ● π-donor H-Bond Tyr402 (7.14 Å)	● π-anion Glu278 (7.81 Å)	● Alkyl Ile149 (5.04 Å)	● VdW Glu119, Lys150, Arg156, Ser180, Ile223	● Donor-Donor clashes Arg118 (4.67 Å) Asp151 (4.04 Å) Arg368 (5.92 Å)
4	trans-Traumatic acid	● Conventional H-Bond Arg152 (4.15 Å) Ile436 (6.00 Å) Thr438 (4.11 Å)	N.D.	● Alkyl Arg118 (4.28, 4.33 Å)	● VdW Glu119, Leu134, Gln136, Ser145, Gly147, Thr148, Ile149, Lys150, Asp151, Arg156, Trp179, Arg430, Trp437	N.D.
5	Azelaic acid	● Conventional H-Bond Arg152 (4.64 Å) Arg368 (6.71 Å) Arg293 (6.24, 6.38 Å)	N.D.	● Alkyl Lys150 (4.80 Å)	● VdW Arg118, Glu119, Ile149, Asp151, Trp179, Ser180, Glu228, Glu278, Tyr402	N.D.
6	Gallic acid	● Conventional H-Bond Glu119 (5.37 Å) Asp151 (3.10 Å) Arg156 (3.33 Å) Glu228 (2.56 Å)	N.D.	● π-alkyl Lys150 (5.17 Å) Arg152 (5.23 Å)	● VdW Arg118, Leu134, Trp179, Ser180, Arg225	N.D.
7	D-arabinonic acid	● Conventional H-Bond Arg118 (4.98 Å) Arg293 (6.54 Å) Arg368 (7.05 Å) Tyr402 (5.12 Å) Ile149 (5.82, 5.90 Å) ● Carbon H-Bond Glu119 (5.48 Å)	● π-anion Arg118 (5.41 Å)	N.D.	● VdW Lys150, Asp151, Glu278	N.D.
8	Pyrogallol	● Conventional H-Bond Arg293 (6.37 Å) Arg368 (6.42 Å) ● π-donor H-Bond Tyr402 (6.29 Å)	● π-anion Glu278 (8.43 Å)	N.D.	● VdW Ile149, Lys150	● Donor-Donor clashes Arg118 (5.18 Å) Arg368 (6.01 Å)
9	D-sorbitol	● Conventional H-Bond Arg118 (6.40 Å) Asp151 (4.99 Å) ● Carbon H-Bond Ile149 (6.79 Å) Lys150 (3.89 Å)	N.D.	N.D.	● VdW Glu119, Arg293, Gly345, Tyr402	● Donor-Donor clashes Arg118 (4.13, 4.98 Å) Arg368 (5.59, 5.73 Å)
10	D-mannitol	● Conventional H-Bond Glu119 (5.14 Å) Ile149 (5.01 Å) Arg293 (6.50 Å) Tyr402 (5.48 Å)	N.D.	N.D.	● VdW Leu134, Lys150, Asp151, Arg156	● Donor-Donor clashes Arg118 (5.44 Å) Arg368 (6.19 Å)
11	DL-Malic acid	● Conventional H-Bond Arg118 (4.77, 6.02 Å) Glu119 (4.70 Å) Arg293 (6.36 Å) Arg368 (6.78 Å) Tyr402 (5.42 Å)	N.D.	N.D.	● VdW Lys150, Glu278	● Donor-Donor clashes Arg368 (5.64 Å)
12	Protocatechuic acid	● Conventional H-Bond Arg152 (3.00 Å) Trp179 (2.87 Å) Ser180 (2.70 Å) Thr226 (2.71 Å) Glu228 (2.23 Å)	● π-anion Glu278 (4.02 Å)	● π-sigma Arg225 (3.68 Å) ● π-alkyl Arg225 (4.30 Å)	● VdW Lys150	● Acceptor-Acceptor clashes Glu227 (2.82 Å)
13	(-)-Quinic acid	● Conventional H-Bond Arg152 (4.04 Å)	N.D.	N.D.	● VdW Arg118, Glu119, Lys150, Arg156, Ser180	● Acceptor-Acceptor clashes Trp179 (4.75 Å) ● Donor-Donor clashes Asp151 (3.96 Å)
14	Shikimic acid	● Conventional H-Bond Arg118 (6.36 Å) Glu119 (5.80 Å) Arg368 (6.13, 6.90 Å) Tyr402 (4.98 Å)	N.D.	N.D.	● VdW Ile149, Lys150, Glu278	● Donor-Donor clashes Arg293 (6.33 Å)
15	3-Methoxybenzoic acid	● Conventional H-Bond Arg368 (3.55 Å) Lys432 (4.91 Å)	N.D.	● Alkyl Arg368 (3.47 Å) ● π-sigma Arg368 (4.42 Å) ● π-alkyl Ile427 (4.59 Å) Pro431 (5.82 Å) Lys432 (4.51 Å)	● VdW Ser367, Trp399, Ser400, Arg428, Gly429, Arg430	N.D.
16	4′-hydroxyacetophenone	● Carbon H-Bond Pro431 (4.82 Å)	● π-cation Lys432 (4.14 Å)	● π-alkyl Arg368 (4.73 Å) Pro431 (4.99 Å)	● VdW Ser367, Trp399, Ile427, Arg428, Gly429, Arg430, Thr438	● Donor-Donor clashes Arg368 (3.69 Å)
17	Salicylic acid	● Conventional H-Bond Arg368 (3.55, 4.98, 5.60 Å) Asn369 (4.21 Å) Lys432 (4.03, 4.77 Å) ● Carbon H-Bond Ser367 (4.14 Å)	N.D.	● π-sigma Arg368 (3.87 Å) ● π-alkyl Ile427 (5.46 Å) Pro431 (5.96 Å) Lys432 (4.30 Å)	● VdW Trp399, Ser400	N.D.
18	Benzoic acid	N.D.	N.D.	● π-sigma Arg368 (3.56 Å) ● π-alkyl Arg368 (4.26 Å) Ile427 (4.89 Å) Lys432 (4.89, 4.96 Å)	● VdW Ser367, Asn369, Trp399, Ser400	N.D.
Control ligand: Natural substrate						
1	Sialic acid	● Conventional H-Bond Arg118 (6.40 Å) Ile149 (5.50 Å) Asp151 (4.36 Å) Arg152 (4.04 Å) Arg156 (5.86 Å) Trp179 (5.67 Å) Tyr402 (5.78 Å)	N.D.	N.D.	● VdW Glu119, Leu134, Thr148, Lys150, Ser180, Arg225, Glu228, Arg293, Arg368	● Donor-Donor clashes Arg118 (4.42 Å)
Control ligand: anti-influenza agents						
1	GJT	● Conventional H-Bond Asp151 (4.80 Å) Arg152 (4.40 Å) Tyr402 (5.92 Å) ● Carbon H-Bond Glu119 (6.21 Å)	N.D.	● Alkyl Arg225 (463 Å) ● π-alkyl Lys150 (4.69 Å)	● VdW Arg118, Thr148, Ile149, Arg156, Trp179, Ser180, Ile223, Glu228, Ser247, Glu277, Glu278, Asn295, Gly345, Val346	● Donor-Donor clashes Arg293 (5.64 Å) Arg368 (6.41 Å)
2	Laninamivir	● Conventional H-Bond Arg118 (6.53 Å) Glu119 (4.96 Å) Arg152 (4.12 Å) Arg368 (6.42, 6.46 Å) ● Carbon H-Bond Glu277 (5.44 Å) Glu278 (6.83 Å) Lys150 (4.19 Å)	N.D.	● Alkyl Ile223 (6.21 Å)	● VdW Leu134, Ile149, Arg156, Trp179, Ser180, Arg225, Ser247, Arg293,Asn295, Tyr402	● Donor-Donor clashes Asp151 (4.31 Å)
3	Peramivir	● Conventional H-Bond Arg118 (6.44 Å) Glu119 (5.60 Å) Arg152 (4.49 Å) Trp179 (5.52, 6.07 Å) Arg293 (6.17 Å) Arg368 (7.03 Å) ● Carbon H-Bond Arg152 (3.60 Å)	N.D.	N.D.	● VdW Leu134, Lys150, Asp151, Arg156, Ser180, Ile223, Arg225, Glu228, Ser247, Glu277, Glu278, Tyr402	● Donor-Donor clashes Arg368 (5.47 Å)
4	Oseltamivir (Tamifilu^®^)	● Conventional H-Bond Glu119 (5.58, 6.27 Å) Arg152 (4.08 Å) Arg293 (6.89 Å) Arg368 (6.23, 6.93 Å) ● Carbon H-Bond Glu278 (6.60 Å)	N.D.	N.D.	● VdW Arg118, Lys150, Trp179, Ser180, Ile223, Arg225, Ser246, Glu277, Asn295, Tyr402	N.D.
5	DANA	● Conventional H-Bond Arg152 (4.06 Å) Glu278 (6.32 Å) Arg293 (5.27 Å) Arg368 (6.30 Å) ● Carbon H-Bond Glu278 (6.74 Å)	N.D.	N.D.	● VdW Arg118, Glu119, Lys150, Ser180, Glu277, Ser247, Gly345, Tyr402	● Donor-Donor clashes Arg225 (4.40 Å) Arg293 (4.63 Å) Arg368 (6.12 Å)
6	Zanamivir (GANA)	● Conventional H-Bond Arg118 (5.26 Å) Glu119 (4.83 Å) Ile149 (4.65 Å) Arg152 (4.34 Å) Trp179 (4.84, 5.85 Å) Glu278 (5.03 Å) Tyr402 (6.58 Å) ● Carbon H-Bond Glu119 (6.73 Å)	N.D.	N.D.	● VdW Leu134, Lys150, Asp151, Arg156, Ser180, Ile223, Arg225, Glu228, Ser247, Glu277, Arg293, Asn295	N.D.
Control ligands: anti-influenza drug derivatives						
1	Oseltamivir-SO2NH-(CH2)2-anthrapyrazole	● Conventional H-bond Glu119 (4.34 Å), Lys432 (5.15 Å), Arg368 (3.92, 6.73 Å), Tyr402 (6.24 Å)	● π-cation Arg368 (4.11, 4.14, 5.46 Å)	● Alkyl Arg225 (4.81 Å) ● π-alkyl Ile427 (6.45 Å) Lys432 (4.74 Å) Pro431 (5.56, 5.73, 6.52 Å) ● π-sigma Arg368 (3.37 Å)	● VdW Arg118, Leu134, Ile149, Lys150, Asp151, Arg152, Arg156, Trp179, Ser180, Ile223, Glu228, Glu278, Arg293, Pro326, Ser367	N.D.
2	Zanamivir-SO2NH-(CH2)2-anthrapyrazole	● Conventional H-bond Arg118 (6.07 Å) Glu119 (4.54 Å) Arg152 (4.07 Å) Arg293 (6.34 Å) Tyr402 (6.20 Å) Lys432 (5.47 Å) Arg 368 (6.14, 6.74 Å) ● Carbon H-bond Glu119 (6.46 Å) Glu278 (6.50 Å) Ile149 (4.60 Å)	● π-cation Arg368 (4.79, 5.78, 5.72 Å) ● Attractive Charges Glu119 (6.60 Å)	● π-alkyl Ile149 (5.11 Å) Pro431 (5.40, 5.91, 6.08 Å) ● π-sulfur Tyr402 (6.20 Å)	● VdW Leu134, Lys150, Asp151, Arg156, Trp179, Ser180, Arg225, Glu228, Ser247, His275, Glu277, Asn295, Asn344, Ser367, Ile427	● Donor-Donor clashes Arg293 (5.01 Å)

N.D.: Not detected.

## Data Availability

All data supporting the conclusions of this article are included in this article.

## Supplementary Materials

The following supporting information can be downloaded at: https://www.mdpi.com/article/10.3390/foods13010081/s1, Table S1: Raw data of LC-MS/MS analysis of *C. mimosoides* aqueous extract; Table S2: Comparing the binding site of molecular docking between NA and HA; Figure S1: Molecular docking of hemagglutinin (HA) (PDB:1RU7) with Oseltamivir using CB dock.
