# Supplementary material for: Unveiling the Potent Antiviral and Antioxidant Activities of an Aqueous Extract from Caesalpinia mimosoides Lamk: Cheminformatics and Molecular Docking Approaches
Source: Foods. 2023 Dec 25;13(1):81. doi: 10.3390/foods13010081 (PMC10778375; doi:10.3390/foods13010081)
Supplement: Supplementary file 1 [file foods-13-00081-s001.zip › foods-2724566-supplementary.pdf]

**Subplimentary material**

**Table S1.** Raw data of LC-MS/MS analysis of *C. mimosoides* aqueous extract

| Metabolites | Analyte Peak Name | Exp.RT/RT (minute) | Peak area | % PeakArea |
|---|---|---|---|---|
| Fenuron | 162.8404 / 0.55 | 0.55/0.58 | 4.44E+05 | 0.43 |
| 2,4-Dichloro-5-methylphenol | 174.8286 / 0.59 | 0.59/0.60 | 7.21E+04 | 0.07 |
| DL-Malic acid | 132.8681 / 0.71 | 0.71/0.80 | 6.03E+05 | 0.58 |
| Salicylic acid | 136.8629 / 0.71 | 0.71/0.88 | 1.97E+05 | 0.19 |
| Digalacturonic acid | 369.0684 / 0.71 | 0.71/0.75 | 1.05E+05 | 0.10 |
| L(+)-Arabinose | 149.0460/ 0.95 $M-H^-$ | 0.95/0.95 | 9.49E+04 | 0.09 |
| N-Methyl-L-leucine | 144.0674/ 0.75 | 0.75/0.78 | 3.44E+05 | 0.33 |
| D-Arabinonic acid | 165.0415/ 0.75 | 0.75/0.77 | 1.69E+05 | 0.16 |
| D-Sorbitol | 181.0728/ 0.75 $M-H^-$ | 0.75/0.75 | 2.47E+05 | 0.24 |
| 5-Keto-D-gluconic acid | 193.0369/ 0.75 | 0.75/0.76 | 8.99E+05 | 0.87 |
| 3-Hydroxy-2',5'-dichlorobiphenyl | 237.0628/ 0.79 | 0.79/0.80 | 5.91E+04 | 0.06 |
| (-)-Quinic acid | 191.0599/ 0.87 | 0.87/0.88 | 1.30E+07 | 12.52 |
| DL-Malic acid | 133.0147/ 1.03 | 1.04/1.05 | 1.68E+05 | 0.16 |
| Salicylic acid | 137.0251/ 1.13 | 1.13/1.14 | 4.12E+05 | 0.40 |
| Shikimic acid | 173.0473/ 1.13 | 1.13/1.14 | 3.77E+06 | 3.62 |
| Pipecolinic acid | 128.0358 / 2.65 | 2.65/2.68 | 4.78E+04 | 0.05 |
| 2,2-Dimethylglutaric acid | 159.0308 / 2.68 | 2.69/2.73 | 2.51E+05 | 0.24 |
| 1,2,3-Benzenetriol (Pyrogallol) | 125.0258 / 2.92 | 2.92/2.93 | 7.78E+06 | 7.49 |
| Gallic acid | 169.0217 / 2.92 | 2.92/2.93 | 3.97E+07 | 38.21 |
| Protocatechuic acid | 153.0199 / 3.53 | 3.53/3.51 | 1.15E+05 | 0.11 |
| Adipic acid | 145.0511 / 3.53 $M-H^-$ | 3.53/3.41 | 2.43E+05 | 0.23 |
| Bergeninum | 327.0742 / 3.68 $M-H^-$ | 3.69/3.70 | 1.41E+06 | 1.36 |
| Bergeninum | 363.0504 / 3.68 $M+Cl^-$ | 3.69/3.70 | 5.84E+05 | 0.56 |
| DL-3,4-Dihydroxyphenyl glycol | 321.0266 / 3.89 $MH_2O-H^-$ | 3.89/3.85 | 8.03E+05 | 0.77 |
| DL-3,4-Dihydroxyphenyl glycol | 339.0372 / 2.92 $M-H^-$ | 2.92/2.93 | 1.86E+05 | 0.18 |

| | | | | |
|---|---|---|---|---|
| (±)-Isorhynchophylline | 383.1932 / 4.01 | 4.01/4.01 | 3.39E+05 | 0.33 |
| Benzoic acid | 121.0662 / 4.13 | 4.13/4.08 | 5.68E+05 | 0.55 |
| 4-Hydroxyacetophenone | 135.0821 / 4.13 | 4.13/4.09 | 7.23E+05 | 0.70 |
| 1,2-Benzenedicarboxylic acid | 165.0926 / 4.13 M-H$^{-}$ | 4.13/4.06 | 5.48E+05 | 0.53 |
| 7-Hydroxychromanone | 163.0771 / 4.13 MH$_2$O-H$^{-}$ | 4.13/4.09 | 1.03E+06 | 0.99 |
| Salicylic acid | 137.0974 / 4.17 | 4.17/4.08 | 1.97E+05 | 0.19 |
| Azelaic acid | 187.0982 / 4.29 | 4.29/4.30 | 1.74E+05 | 0.17 |
| Betulinic acid | 455.2500 / 4.33 | 4.33/4.31 | 8.73E+05 | 0.84 |
| 4-Pregnen-17.alpha., 20.beta.-diol-3-one-20-sulfate | 411.2243/ 4.33 | 4.33/4.31 | 1.08E+06 | 1.04 |
| 3-Methoxybenzoic acid | 151.0403/ 4.40 | 4.40/4.39 | 6.12E+04 | 0.06 |
| trans-Traumatic acid | 227.2028 / 4.45 | 4.45/4.58 | 1.64E+05 | 0.16 |
| 2-Mercaptobenzothiazole | 165.9796 / 4.48 | 4.48/4.49 | 2.99E+05 | 0.29 |
| 1,11-Undecanedicarboxylic acid | 243.1614 / 4.52 | 4.52/4.38 | 2.60E+05 | 0.25 |
| 3-Hydroxy-4-methoxycinnamic acid | 193.0879 / 4.56 | 4.56/4.64 | 1.05E+05 | 0.10 |
| 1-Naphthol | 143.1080 / 4.60 | 4.60/4.62 | 1.10E+05 | 0.11 |
| Benzamide, N-hydroxy-3-3-(hydroxyamino)-3-oxo-1-propen-1-yl | 221.1554 / 4.60 | 4.60/4.61 | 1.13E+05 | 0.11 |
| Cefuroxime | 422.9780 / 4.61 | 4.61/5.00 | 3.19E+05 | 0.31 |
| 5-Chloroindole-2-carboxylic acid | 194.0837 / 4.72 | 4.72/4.71 | 2.06E+06 | 1.99 |
| Hexadecanedioic acid | 285.2080 / 4.76 | 4.76/4.73 | 1.71E+05 | 0.16 |
| 9-Oxo-11R,15S,19R-trihydroxyprostanoic acid | 371.2598 / 4.76 | 4.76/4.77 | 2.53E+05 | 0.24 |
| Tetradecanedioic acid | 257.1775 / 4.83 | 4.84/4.85 | 5.54E+05 | 0.53 |
| Mono-2-ethylhexyl phthalate | 277.1456 / 4.83 | 4.84/5.00 | 3.66E+05 | 0.35 |
| Isorhamnetin | 315.2534 / 4.83 | 4.84/4.85 | 1.23E+06 | 1.19 |
| 9,10-Dihydroxy-12Z-octadecenoic acid | 313.1821 / 4.87 | 4.87/4.98 | 3.79E+05 | 0.37 |
| Heptadecanoic acid | 269.2138 / 4.99 | 4.99/5.01 | 6.70E+05 | 0.65 |
| Dodecylbenzenesulfonic acid | 325.1851 / 4.99 | 4.99/5.02 | 1.86E+06 | 1.79 |
| 2-Methoxyestradiol | 301.2215 / 5.07 | 5.07/5.04 | 1.08E+06 | 1.04 |
| Diisodecyl phthalate | 305.1765 / 5.07 | 5.07/5.04 | 1.02E+05 | 0.10 |
| 3,4'-Dimethoxy-2-hydroxychalcone | 283.2650 / 5.15 | 5.15/5.11 | 7.08E+05 | 0.68 |
| 16-Hydroxyhexadecanoic acid | 271.2289 / 5.19 | 5.19/5.08 | 6.34E+05 | 0.61 |
| 3-Hydroxyoctadecanoic acid | 299.2046 / 5.24 M H$_2$O-H$^{-}$ | 5.24/5.27 | 2.60E+06 | 2.50 |

| | | | | |
|---|---|---|---|---|
| Dodecyl sulfate | 265.1498 / 5.37 | 5.37/5.40 | 1.17E+07 | 11.26 |
| 2-Naphthalenethiol | 158.8469 / 5.84 | 5.84/5.86 | 2.80E+05 | 0.27 |
| 3-Hydroxy-4-methoxybenzoic acid | 166.8339 / 5.84 | 5.84/5.89 | 2.43E+05 | 0.23 |
| 9,10-Dihydroxy-12Z-octadecenoic acid | 312.9009 / 6.80 $M-H^-$ | 6.80/6.38 | 3.29E+05 | 0.32 |

**Table S2.** Comparing the binding site of molecular docking between NA and HA.

| Compounds | Residues polar interaction with sialic acid cavity | | | | | | | | | | | | PDB |
|---|---|---|---|---|---|---|---|---|---|---|---|---|---|
| | - | Glu119 | Arg152 | Arg156 | - | Ser179 | Glu227 | Glu276 | Glu277 | Arg292 | Arg371 | Tyr406 | 3CKZ |
| | Arg118 | Glu119 | Arg152 | Arg156 | Trp179 | Ser180 | Glu228 | Glu277 | Glu278 | Arg293 | Arg368 | Tyr402 | **6HP0** |
| **Dicaboxylic acids** | | | | | | | | | | | | | |
| Azelaic acid | Arg118 | Glu119 | Arg152 | Arg156 | Trp179 | - | - | - | Glu278 | Arg293 | Arg368 | Tyr402 | |
| DL-Malic acid | Arg118 | Glu119 | - | - | - | - | - | - | Glu278 | Arg293 | Arg368 | Tyr402 | |
| Hexadecanedioic acid | Arg118 | Glu119 | Arg152 | Arg156 | Trp179 | Ser180 | Glu228 | Glu277 | Glu278 | Arg293 | - | Tyr402 | |
| Tetradecanedioic acid | Arg118 | Glu119 | Arg152 | Arg156 | - | Ser180 | Glu228 | - | Glu278 | - | - | - | |
| trans-Traumatic acid | Arg118 | Glu119 | Arg152 | Arg156 | Trp179 | Ser180 | - | - | - | - | - | - | |
| **Sugar derivatives** | | | | | | | | | | | | | |
| D-arabinonic acid | Arg118 | Glu119 | - | - | - | - | - | - | Glu278 | Arg293 | Arg368 | Tyr402 | |
| D-mannitol | Arg118 | Glu119 | - | Arg156 | Trp179 | - | - | - | Glu278 | Arg293 | Arg368 | Tyr402 | |
| D-sorbitol | Arg118 | Glu119 | - | - | - | - | - | - | Glu278 | Arg293 | Arg368 | Tyr402 | |
| **Phenolic acids** | | | | | | | | | | | | | |
| Gallic acid | Arg118 | Glu119 | Arg152 | Arg156 | Trp179 | Ser180 | Glu228 | - | - | - | - | - | |
| Protocatechuic acid | - | - | Arg152 | - | Trp179 | Ser180 | Glu228 | Glu277 | Glu278 | - | - | - | |
| Pyrogallol | Arg118 | Glu119 | - | - | - | - | - | - | Glu278 | Arg293 | Arg368 | Tyr402 | |
| (-)-Quinic acid | - | Glu119 | Arg152 | - | Trp179 | Ser180 | Glu228 | Glu277 | Glu278 | - | - | Tyr402 | |
| Shikimic acid | Arg118 | Glu119 | - | - | - | - | Glu228 | - | Glu278 | Arg293 | Arg368 | Tyr402 | |
| **Meyhylated analogs** | | | | | | | | | | | | | |
| Berginin | Arg118 | Glu119 | Arg152 | Arg156 | Trp179 | Ser180 | Glu228 | - | Glu278 | Arg293 | Arg368 | Tyr402 | |
| **Control ligand: Natural substrate** | | | | | | | | | | | | | |
| Sialic acid | Arg118 | Glu119 | Arg152 | Arg156 | Trp179 | Ser180 | Glu228 | - | Glu278 | Arg293 | Arg368 | Tyr402 | |
| **Control ligand: antineuraminidase drugs** | | | | | | | | | | | | | |
| DANA | Arg118 | Glu119 | Arg152 | Arg156 | Trp179 | Ser180 | Glu228 | - | Glu278 | Arg293 | Arg368 | Tyr402 | |
| GJT | Arg118 | Glu119 | Arg152 | Arg156 | Trp179 | Ser180 | Glu228 | Glu277 | Glu278 | Arg293 | Arg368 | Tyr402 | |
| Laninamivir | Arg118 | Glu119 | Arg152 | Arg156 | Trp179 | Ser180 | Glu228 | Glu277 | Glu278 | - | - | - | |
| Oseltamivir (Tamifilu®) | Arg118 | Glu119 | Arg152 | - | Trp179 | Ser180 | Glu228 | Glu277 | Glu278 | Arg293 | Arg368 | Tyr402 | |
| Peramivir | Arg118 | Glu119 | Arg152 | Arg156 | Trp179 | Ser180 | Glu228 | Glu277 | Glu278 | Arg293 | Arg368 | Tyr402 | |
| Zanamivir (GANA) | Arg118 | Glu119 | Arg152 | Arg156 | Trp179 | Ser180 | Glu228 | Glu277 | Glu278 | Arg293 | Arg368 | Tyr402 | |

| Compounds | Residues polar interaction with 430-cavity | | | | | | | | | PDB |
|---|---|---|---|---|---|---|---|---|---|---|
| | Pro326 | - | - | Ile427 | - | - | - | - | Thr439 | **3CKZ[1]** |
| | Pro326 | Arg368 | Asn369 | Ile427 | Arg430 | Pro431 | Lys432 | Glu433 | Thr439 | **6HP0[2]** |
| **Phenolic acids** | | | | | | | | | | |
| Benzoic acid | - | Arg368 | Asn370 | Ile427 | Arg430 | Pro431 | Lys432 | - | - | |
| Salicylic acid | - | Arg368 | Asn374 | Ile427 | Arg430 | Pro431 | Lys432 | - | - | |
| **Meyhylated analogs** | | | | | | | | | | |
| 3-Hydroxy-4-methoxycinnamic acid | Pro326 | Arg368 | Asn371 | Ile427 | Arg430 | Pro431 | Lys432 | Glu433 | - | |
| 3-Methoxybenzoic acid | - | Arg368 | Asn372 | Ile427 | Arg430 | Pro431 | Lys432 | Glu433 | - | |
| 4′-hydroxyacetophenone | - | Arg368 | Asn373 | Ile427 | Arg430 | Pro431 | Lys432 | - | Thr438 | |

**Figure S1.** Molecular docking of hemagglutinin (HA) (PDB:1RU7) with Oseltamivir using CB dock.

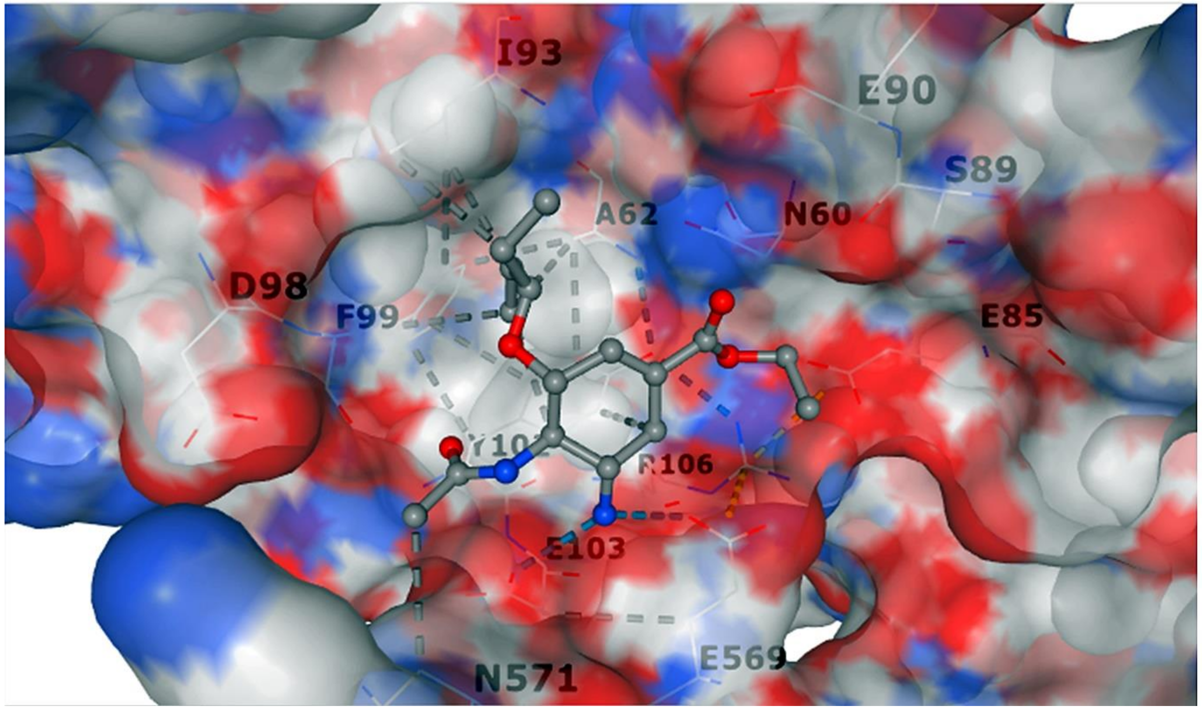


| CurPocket ID | Vina score | Cavity volume (Å$^3$) | Center (x, y, z) | Docking size (x, y, z) | Contact residues |
|---|---|---|---|---|---|
| ○C5 | -6.3 | 260 | 39, 78, 97 | 21, 21, 21 | View |
| ⊙C1 | -5.4 | 750 | 37, 81, 81 | 21, 21, 21 | View |
| ○C3 | -5.1 | 473 | 44, 88, 141 | 21, 21, 21 | View |
| ○C4 | -5.1 | 282 | 42, 95, 92 | 21, 21, 21 | View |
| ○C2 | -4.8 | 667 | 36, 92, 106 | 21, 21, 21 | View |

**Pocket: C1 & Score: -5.4**

**Chain A**: ASN60 ALA62 GLU85 SER89 GLU90 ILE93 ASP98 PHE99 TYR102 GLU103 ARG106
